# MaizeCODE reveals bi-directionally expressed enhancers that harbor molecular signatures of maize domestication

**DOI:** 10.1038/s41467-024-55195-w

**Published:** 2024-12-30

**Authors:** Jonathan Cahn, Michael Regulski, Jason Lynn, Evan Ernst, Cristiane de Santis Alves, Srividya Ramakrishnan, Kapeel Chougule, Sharon Wei, Zhenyuan Lu, Xiaosa Xu, Umamaheswari Ramu, Jorg Drenkow, Cassidy Danyko, Melissa Kramer, Arun Seetharam, Matthew B. Hufford, W. Richard McCombie, Doreen Ware, David Jackson, Michael C. Schatz, Thomas R. Gingeras, Robert A. Martienssen

**Affiliations:** 1 Howard Hughes Medical Institute, Cold Spring Harbor Laboratory, 1 Bungtown Road, Cold Spring Harbor, NY 11724 USA; 2 Cold Spring Harbor Laboratory, 1 Bungtown Road, Cold Spring Harbor, NY 11724 USA; 3 Johns Hopkins University; 1900 E. Monument Street, Baltimore, MD 21205 USA; 4 Department of Ecology, Evolution, and Organismal Biology, Iowa State University, Ames, IA 50011 USA; 5 USDA ARS Robert W. Holley Center for Agriculture and Health Cornell University, Ithaca, NY USA; 6 Present Address: Department of Plant Biology, University of California, Davis, CA 95616 USA

**Keywords:** Epigenomics, Histone post-translational modifications, Plant domestication, Transcriptional regulatory elements

## Abstract

Modern maize (*Zea mays* ssp. *mays*) was domesticated from *Teosinte parviglumis* (*Zea mays* ssp. *parviglumis*), with subsequent introgressions from *Teosinte mexicana* (*Zea mays* ssp. *mexicana*), yielding increased kernel row number, loss of the hard fruit case and dissociation from the cob upon maturity, as well as fewer tillers. Molecular approaches have identified transcription factors controlling these traits, yet revealed that a complex regulatory network is at play. MaizeCODE deploys ENCODE strategies to catalog regulatory regions in the maize genome, generating histone modification and transcription factor ChIP-seq in parallel with transcriptomics datasets in 5 tissues of 3 inbred lines which span the phenotypic diversity of maize, as well as the teosinte inbred TIL11. Transcriptomic analysis reveals that pollen grains share features with endosperm, and express dozens of “proto-miRNAs” potential vestiges of gene drive and hybrid incompatibility. Integrated analysis with chromatin modifications results in the identification of a comprehensive set of regulatory regions in each tissue of each inbred, and notably of distal enhancers expressing non-coding enhancer RNAs bi-directionally, reminiscent of “super enhancers” in animal genomes. Furthermore, the morphological traits selected during domestication are recapitulated, both in gene expression and within regulatory regions containing enhancer RNAs, while highlighting the conflict between enhancer activity and silencing of the neighboring transposable elements.

## Abstract (web summary)

MaizeCODE maps active regulatory regions tied to maize domestication traits in a diverse panel of tissues and inbreds. It reveals bi-directional enhancer RNAs and the molecular conflicts between activity and silencing of non-coding regions.

## Introduction

Modern maize (*Zea mays* ssp. *mays*) is the result of domestication from its ancestor teosinte *parviglumis* (*Zea mays* ssp. *parviglumis*), with subsequent introgressions from teosinte *mexicana* (*Zea mays* ssp. *mexicana*)^1–6^. Domestication traits include increasing the number of kernels per ear, limiting tillering, removing the hard fruitcase and preventing the kernels from disarticulating upon maturation^3^. The genetic study of the domestication process has led to the identification of many key regulators, mostly transcription factors (TFs), responsible for some of these traits. The most important of these regulatory genes is *TEOSINTE BRANCHED (TB1)*, which encodes a TF with a basic helix-loop-helix DNA binding domain^7,8^ that defines the TCP family of TFs (TEOSINTE BRANCHED, CYCLOIDEA, PROLIFERATING CELL NUCLEAR ANTIGEN FACTOR)^9^. TB1 is a master-regulator, regulating other TFs as well as itself, in a tissue-specific manner^8,10^. Among its targets, *GRASSY TILLERS 1 (GT1)* promotes apical dominance along with TB1 in modern maize^11^. Several other genes have been implicated in the domestication or improvement processes, such as *TUNICATE 1 (TU1/ZMM19)*^12,13^, *RAMOSA1 (RA1)*^14,15^ and *TEOSINTE GLUME ARCHITECTURE (TGA1)*^16^ but it is now clear that a complex epistatic network of genes has evolved through domestication, which relies more on quantitative regulation than presence/absence^2–4^.

Identification of regulatory regions has been pioneered in animal genomes by the ENCODE (Encyclopedia of DNA elements) project^17,18^. ENCODE relies on integrating datasets which evaluate chromatin structure, such as measuring DNA accessibility and histone post-translational modifications, with transcriptomic datasets to identify regions of the genome that could regulate and/or register gene expression. In animals, distal regulatory regions, also called enhancers, are usually marked by mono-methylation of lysine 4 on the histone H3 tail (H3K4me1), while active enhancers are associated with histone H3 acetylation (H3ac) and inactive enhancers are enriched for H3K27me3^19^. Clusters of regulatory regions known as “super-enhancers” have additional signatures, notably the presence of capped RNA molecules called enhancer RNAs, which are transcribed from both strands of DNA in these distal regulatory regions^20–22^.

The study of *cis*-regulatory regions in plants have revealed similar molecular signatures^23^, except that H3K4me1 is found in gene bodies rather than distal enhancers^10,24–26^, and the presence of enhancer RNAs is disputed^27^. The catalog of distal elements in maize has been greatly improved by recent efforts to resolve cell-type specific accessible regions with single-cell ATAC-seq experiments^28^. ATAC-seq identifies nucleosome free and other open chromatin regions, which include many but not all promoters and enhancers, as well as many other regions of the genome accessible to bacterial transposase. While ATAC-seq is uniquely powerful in the single cell context, open chromatin alone does not generate a comprehensive dataset of active regulatory regions^29^, especially when limited to selected tissues and inbred lines.

By careful selection of tissues, inbred lines, and epigenomic signatures we present MaizeCODE, a comprehensive catalog of active maize *cis*-regulatory regions, accompanied by a computational pipeline for their analysis. We also present datasets from the teosinte inbred line TIL11, as well as a chromosome level genome sequence assembly, allowing us to investigate the impact of domestication on gene regulation. Through this integrated analysis, we identify tissue-specific enhancers with bi-directionally expressed enhancer RNAs, in each tissue of all inbreds. These “super-enhancers” are more accessible to regulatory TFs and inherently drive higher transcription levels. Interestingly, “super-enhancers” also have stronger RNA-directed DNA methylation (RdDM) signals at their boundaries, including both polIV and polV transcripts. This could reflect the co-evolution or co-regulation of active regulatory regions and the silencing of neighboring TEs. We illustrate the utility of these datasets and their analysis by demonstrating the tissue-specific impact of domestication on the conservation of enhancers and of the genes they regulate. For example, we demonstrate that regulation of ear development was a major target of maize domestication. We also uncover variation in telomere maintenance in pollen and endosperm that could underlie McClintock’s chromosome breakage-fusion-bridge cycle.

## Results

### Reference genome assemblies and data types selected for MaizeCODE

We selected one stiff-stalk (B73), one non-stiff-stalk (W22) and one tropical maize inbred (NC350) to sample the pool of inbreds comprising modern maize, for which high-quality genome sequences and annotations are available^30,31^. We also selected the color converted W22 inbred, which is widely used for transposon mutagenesis, to identify transposon insertions in regulatory and coding regions^32^. We hoped to identify distal regulatory regions that might account for the very high proportion of SNPs that lie in intergenic regions found in Genome Wide Association Studies (GWAS)^10,33^, such as in the Nested Association Mapping population (NAM)^31^. B73 and NC350 are both NAM lines used in these studies. We also generated a high-quality reference genome assembly from the teosinte inbred line TIL11, using PacBio HiFi and BioNano Optical Mapping (“Methods”). An un-scaffolded assembly was published recently, revealing substantial intergenic transposon insertion variation between B73 and TIL11^34^. We subjected our high-quality assembly of TIL11 to the same annotation pipelines as the published maize inbred genomes for consistency (Supplementary Fig. 1a). The TIL11 genome has several megabase-long inversions on chromosomes 1, 2, 4 and 7 relative to all maize inbreds (Supplementary Fig. 1b, c) likely representative of an event predating maize domestication. Furthermore, large differences between inbreds are also present, for example a duplication found only in W22 on chromosome 3, or a duplication found only in NC350 on chromosome 10 (Supplementary Fig. 1c).

Regulatory regions bind transcription factors, which in turn recruit chromatin remodelers and histone modifiers to modify surrounding nucleosomes. Nucleosome free regions can be identified by sensitivity to nucleases, while modification of flanking nucleosomes can be detected by ChIP-seq and define different classes of regulatory regions. MaizeCODE data types conformed to ENCODE standards (“Methods”), but with plant-specific outcomes. Histone marks assessed by ChIP seq were H3K27ac and H3K4me3 (active enhancers and TSS), and H3K4me1 (gene bodies in plants) which complemented differential nuclease sensitivity data (DNS-seq) previously obtained from the same tissues in B73, as part of the MaizeCODE project^35^. RNA datasets included polyA+ RNA-seq, RAMPAGE (5’ caps) and non-coding “short” RNA (or shRNA) of 150nt or less with 5’ tri- or mono-phosphate and 3’ hydroxyl groups. In plants, RAMPAGE and polyA+ RNA-seq includes mRNA and ncRNA products of RNA polymerase II, while shRNA includes products of all 5 RNA polymerases, including 20–24nt short interfering siRNAs (RNA Pol II and Pol IV), and longer transcripts generated by Pol V^36,37^. RNA and chromatin samples were extracted from mature pollen, 5–10 mm immature ears, 1–3 mm root tips, endosperm harvested 15 days after pollination, and coleoptilar nodes (CN) 1 week after germination (Supplementary Table 1; “Methods”).

### H3K27ac marks genes and active enhancers bound by transcription factors

**Fig. 1:**
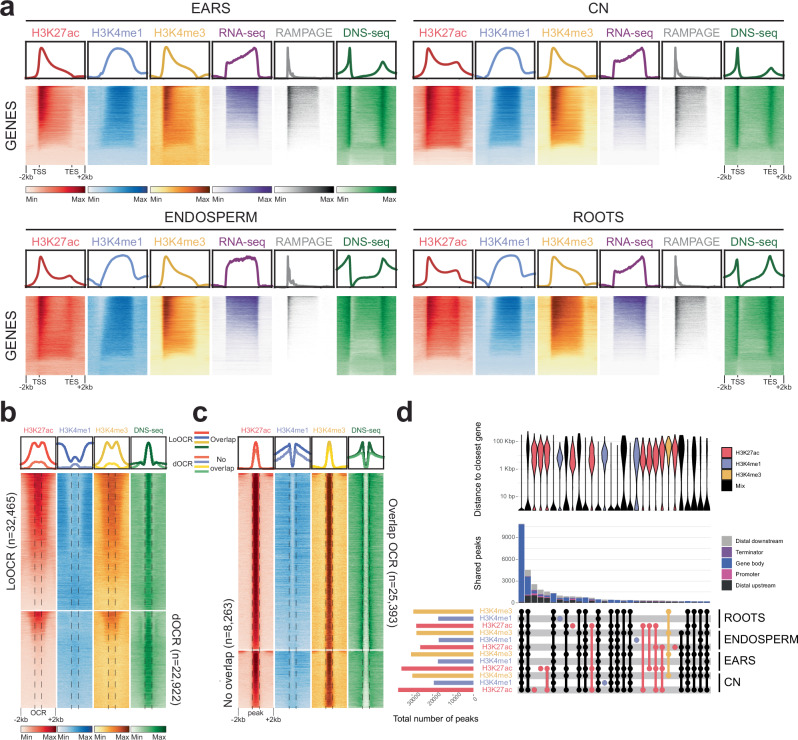
Histone H3 modifications mark DNA regulatory elements in maize inbred lines. **a** Heatmaps and metaplots of H3K27ac, H3K4me1, H3K4me3, RNA-seq, RAMPAGE and differential nucleosome sensitivity (DNS-seq)^35^ over all annotated genes in each tissue of B73 (NAM reference genome), scaled to the same size, with 2 kb upstream and downstream. CN = coleoptilar node. **b** Heatmaps and metaplots of B73 ears H3K27ac, H3K4me1, H3K4me3 and DNS-seq in local and distal open chromatin regions (LoOCR and dOCR, respectively) previously identified by ATAC-seq^38^. Bona fide regulatory elements are enriched for H3K27Ac and H3K4me3 but not H3K4me1. **c** Heatmaps and metaplots of H3K27ac, H3K4me1, H3K4me3 and DNS-seq at all H3K27ac peaks (regulatory elements) in B73 ears. 25,393 peaks intersect previously identified OCRs (20,334 LoOCRs and 5059 dOCRs) but 8263 peaks do not overlap. **d** Summary of shared ChIP-seq peaks in W22 (v2 reference genome). The Upset plot (lower panel) displays the overlap between H3K27ac, H3K4me1 and H3K4me3 peaks in the four tissues. The total number of peaks in each sample is shown on the histogram on the left-hand side of the intersection matrix, while the number of shared peaks between samples is shown above (middle panel), color coded by genomic feature. The violin plot (upper panel) compares the distance between peaks and the closest gene. Tissue specific peaks are mostly at distal elements, whereas loci with several histone marks in multiple tissues are mostly at annotated genes. Distal regulatory elements lie between 2 kb and 100 kb from the nearest gene.

We identified active regulatory regions by integrating H3K4me1, H3K4me3 and H3K27ac ChIP-seq datasets with RNA-seq, RAMPAGE and DNS-seq datasets in four tissues in B73, and with ChIP-seq, RAMPAGE and RNA-seq in the other inbreds (Supplementary Table 1). The profiles of these histone marks over all genes (Fig. 1a) were similar to previously described patterns: peaks of H3K27ac, H3K4me3 and DNS-seq at the TSS, and peaks of H3K4me1 over the gene body^10,26^ (Fig. 1a). These signals were present over transcribed genes, as shown by RAMPAGE and RNA-seq signals in the different tissues tested (Fig. 1a) and in the different inbreds (Supplementary Fig. 2a). We could confirm that these marks are enriched in or near open chromatin regions (OCRs) previously identified by ATAC-seq in ears^38^ (Fig. 1b). The majority of local OCRs (LoOCRs, i.e. promoters and transcription start sites) were marked by H3K27ac and H3K4me3, as expected, while only a subset of distal OCRs (dOCRs) were marked by H3K27ac and H3K4me3, likely corresponding to the active enhancers^10^ (Fig. 1b). Of note, whereas LoOCRs with high H3K27ac and H3K4me3 levels also showed H3K4me1 enrichment within 2 kb up and downstream of the OCR, H3K4me1 was mostly absent from dOCRs, consistent with it exclusively marking gene bodies in plants (Fig. 1b). Regulatory elements are open chromatin regions to which transcription factors (TFs) can bind, and are flanked by acetylated nucleosomes when they are active^10,39^. Consistently, we observed that H3K27ac is deposited at nucleosomes flanking both sides of the OCRs (Fig. 1b; Supplementary Fig. 2b). In addition, some H3K27ac peaks did not overlap OCRs defined by ATAC-seq in younger tissues, despite having similar enrichment values in our ChIP-seq experiments (Fig. 1c). These elements could represent regions controlled by pioneer TFs, which can access DNA even in closed chromatin^40^, and are particularly important for cell-fate transitions^41,42^. They more likely represent an activity specific to a subset of cells in these heterogenous tissues which cannot be detected by nuclease sensitivity earlier in development, or an epigenetic memory of previous activity, yet with potential regulatory function. We thus used H3K27ac peaks to define the boundaries of putative active regulatory regions, whether at promoters and TSS (i.e. LoOCRs), or at distal enhancers (i.e. dOCRs).

We then investigated the tissue-specificity of regulatory regions, by assessing the overlap between H3K27ac, H3K4me1 and H3K4me3 peaks in all tissues of the same inbred. Despite some variability in the number of peaks called in each sample, inherent to both the ChIP-seq methodology and the peak calling algorithm, the largest sets of intersections corresponded to genic regions containing these 3 active marks in all four tissues investigated (Fig. 1d). This approach thus highlighted that most putative regulatory regions are shared between tissues, corresponding to the promoters of constitutively expressed genes. When all 3 modifications were found together, they were mostly found in gene bodies, whereas when only one modification was present, notably H3K27ac, it was found in distal regions. These distal regions were located in a bimodal distribution centered around 2 kb and 50 kb upstream or downstream of the nearest gene (Fig. 1d), reflecting the distribution of transposable elements in the maize genome^25^.

**Fig. 2:**
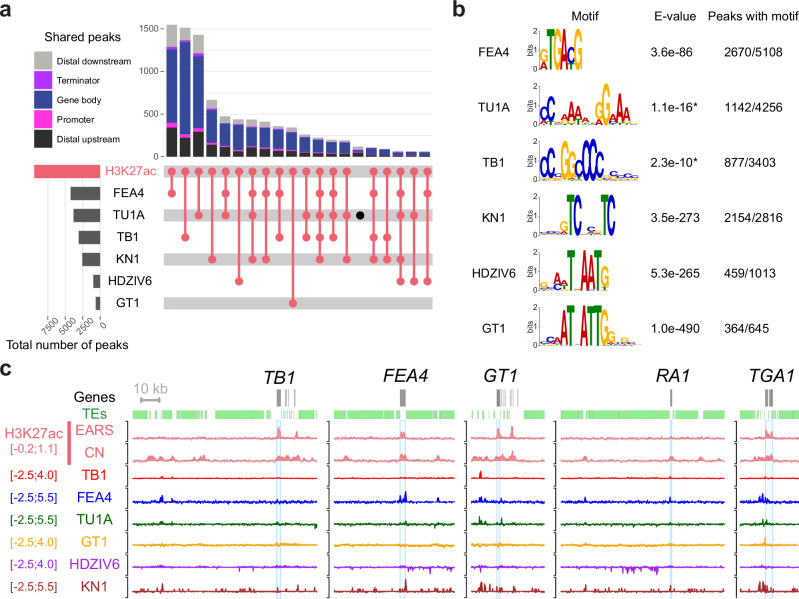
Enhancers at domestication genes are bound by transcription factor networks. **a** Upset plot showing the overlap between H3K27ac peaks identified in B73 and binding sites of six transcription factors (TFBS) analyzed in this study. The total number of peaks called for each sample is shown on the histogram on the left-hand side. The number of shared peaks between the different samples are shown above the intersection matrix, each peak being colored by the genomic feature it intersects with. The majority of the TFBS are indeed within H3K27ac peaks, mostly overlapping gene bodies or at distal regions (>2 kb from a gene) and highlights the interplay between these TFs. **b** Best binding motif identified in each TF peaks with meme or streme (*)^97^. The motifs correspond to the respective family of each TF. **c** Browser screenshots at major domestication loci (*TB1*, *GT1*, *RA1* and *TGA1*) as well as *FEA4*, which regulates a domestication trait, showing complex regulation of these developmental TFs with often co-regulation and auto-regulation.

In addition to histone modifications, we analyzed the binding profiles of selected transcription factors in related tissues to illustrate how MaizeCODE datasets can be used to investigate mechanisms of domestication^8,11,12,43–45^. Almost all the binding sites (TFBS) coincided with H3K27ac peaks in at least one tissue (Fig. 2a). The majority of TFBS were within or close to a gene body, but about a third of the peaks overlapped distal regulatory regions (Fig. 2a). We also observed that each TF had a specific subset of targets, representing their unique regulatory networks (Fig. 2a). This observation was also highlighted by the fact that each TF had a preferential binding motif, which was representative of their family and DNA binding domain (Fig. 2b). Many enhancers contained binding sites for multiple TFs, notably between FASCIATED EAR 4 (FEA4) and TU1-A. Interestingly, TFs often bound their own promoters and promoters of major domestication genes, while distal enhancers at the domestication genes *TB1*, *GT1*, *TGA1* and *RA1* were co-regulated by several TFs (Fig. 2c). These distal enhancers were also active in coleoptilar nodes, which include axillary buds, supporting previously identified branch suppression networks^13,46^. Overall, our analysis shows that H3K27ac peaks correlate well with active regulatory regions, whether marking TSS, proximal or distal enhancers, and harbor binding sites of developmental TFs whose functions have been refined during domestication.

### Pollen has a unique transcription profile for coding regions and small RNAs

**Fig. 3:**
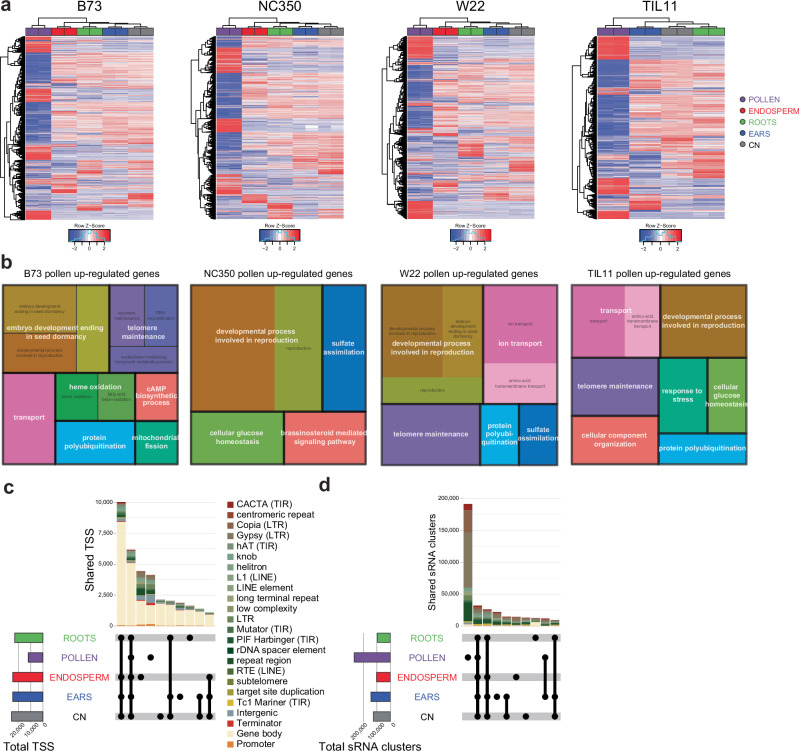
Pollen has a unique transcriptional profile compared to other tissues. **a** Heatmap of all differentially expressed genes (DEGs) in each inbred and their expression level in each tissue (normalized z-score). **b** Gene ontology (GO) terms enriched in genes up-regulated in pollen versus all other tissues for each inbred. NC350 is missing DEGs involved in telomere maintenance. This difference is shared with endosperm (Supplementary Fig. 2) **c** Upset plot of transcription start sites (TSS) identified by RAMPAGE in B73. The total number of TSS in each tissue is shown on the histogram on the left-hand side. The number of shared TSS between the different tissues are shown above the intersection matrix, color coded by genomic feature (including transposable element families). **d** Upset plot of the sRNA clusters identified in shRNA-seq in B73. The total number of clusters in each tissue is shown on the histogram on the left-hand side. The number of shared sRNA clusters between the different tissues are shown above the intersection matrix, color coded by genomic feature (including transposable element families).

In parallel to the profiling of chromatin marks, we performed total RNA-seq in up to five tissues of the four inbreds (Supplementary Table 1). As previously described^47^, our MaizeCODE RNA-seq data revealed that pollen had the most distinct gene expression profile, followed by endosperm (Fig. 3a; Supplementary Fig. 3a). Thousands of genes were differentially expressed (DEGs) between each pair of tissues, with almost 4000 genes up-regulated, and more than 5000 genes down-regulated in pollen versus the four other tissues (Fig. 3a; Supplementary Data 1). By contrast, immature ears had only 260 up- and 191 down-regulated genes versus all other tissues, and coleoptilar nodes had only 21 down-regulated genes compared to the four other tissues (Fig. 3a; Supplementary Data 1). Gene ontology (GO) analysis identified relevant enriched categories in pollen DEGs, including reproductive mechanisms, found in all inbreds (Fig. 3b). Interestingly, genes involved in telomere maintenance were up-regulated in both pollen and endosperm (Fig. 3b; Supplementary Fig. 3b), the two tissues that engage in extended Breakage-Fusion-Bridge (BFB) cycles^48^, presumably due to aberrant telomere healing^49^. However, telomere maintenance genes were not upregulated in NC350 pollen or endosperm, suggesting regulatory variation potentially underlying variation in chromosome healing first described by McClintock^49^. While further experimental evidence is needed to support this hypothesis, NC350 has much longer telomeres than the other inbreds in genome assemblies (Supplementary Fig. 3c), as estimated by the number of times the telomere repeat “CCCTAAA” is found at chromosome ends. These numbers are likely under-estimations, as telomere-to-telomere assembly of Mo17 obtained a larger estimate of 3700 copies per telomere^50^.

In addition to steady-state mRNA levels, we investigated the levels of capped RNA by RAMPAGE, which typically marks Transcription Start Sites (TSS)^51^. Consistent with RNA-seq, pollen had the fewest TSS, about half of those shared between the other tissues (Fig. 3c). The large majority of loci (80%) shared between tissues mapped to annotated genes, but over 50% of the TSS unique to either pollen or endosperm were found in TEs and intergenic regions (Fig. 3c).

**Fig. 4:**
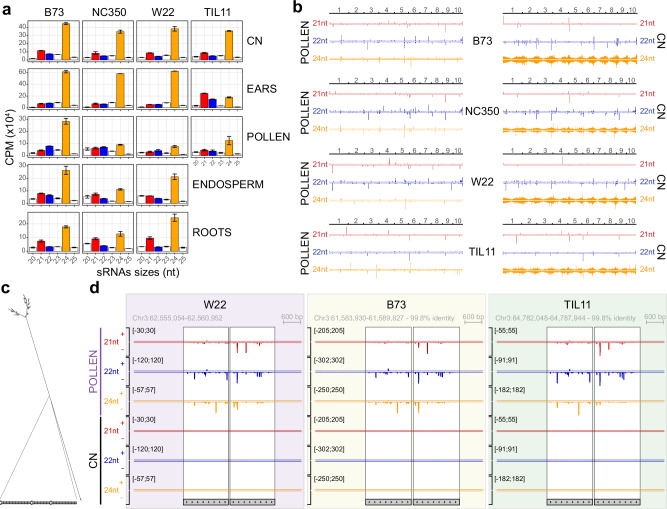
Small RNA size distributions differ among tissues and inbreds. **a** Size distributions of sRNAs were calculated in each tissue of each inbred line (CPM, count per million mapped reads). Maize and teosinte inbreds have similar size distributions in coleoptilar nodes (CN), but differ in other tissues. In pollen, more 24nt sRNA (orange) accumulates in B73 relative to other inbreds, while in ears TIL11 has reduced levels of 24nt and increased levels of 21nt sRNAs (red). In root tips and endosperm, NC350 has reduced levels of 24nt siRNAs. Error bars are standard error between two biological replicates. **b** Whole-genome browsers of 21, 22 and 24nt siRNAs expressed in pollen and coleoptilar node (CN) in each inbred, highlighting the presence of hairpins producing high levels of 22 and 24nt sRNAs in pollen and only 22nt in CN. Each track is scaled to its maximum CPM. At this scale the 21nt sRNAs mostly show expression of the most highly expressed microRNAs. **c** Secondary structure of a representative pollen-specific hairpin made with RNAfold^112^. 50 bp of the 3 kb stem is shown at the bottom. **d** Browser screenshots of a representative pollen-specific hairpin (same as in **c**), present in W22, B73 and TIL11, producing high levels of stranded 22nt sRNAs, as well as 21 and 24nt sRNAs in all pollen inbreds in a very similar pattern. The gray boxes represent the two repeated halves of the hairpin.

Contrastingly, the majority of siRNA clusters identified by short RNA-seq were unique to pollen and mapped to TEs, most notably long terminal repeat (LTR) retrotransposons (Fig. 3d). The second and third largest intersections were composed of clusters shared by all tissues, and shared by all tissues except pollen, respectively (Fig. 3d), further emphasizing the uniqueness of the pollen transcription profile, both coding and non-coding. Analysis of the size distributions of small RNAs revealed that B73 accumulated more 24nt than 21/22nt siRNAs in all tissues (Fig. 4a), but in the other inbreds levels of 24nt and 21/22nt were similar in pollen as previously reported for W22^52^. Intriguingly, immature ears of TIL11 also had much lower levels of 24nt sRNAs, and higher levels of 21/22nt siRNAs, potentially due to differential activity of Dicer-like enzymes in teosinte^53^. We have recently described the presence of multiple non-coding pollen-specific hairpin RNAs on each of the 10 chromosomes of maize inbred W22^52^. Our MaizeCODE data revealed that similar hairpins exist in the other inbreds, and notably in teosinte *parviglumis* (Fig. 4b). These several kilobase-long loci encode stable secondary structures (Fig. 4c), which produce 22nt siRNAs strongly biased to one strand (Fig. 4d), consistent with processing from hairpin precursors by DCL2^52^. Interestingly, we found that 21nt and 24nt siRNAs are also produced from the same sequences in pollen, but not in CN (Fig. 4d), resulting in drastically different whole-genome distributions of 24nt in pollen vs CN in all inbreds (Fig. 4b).

### Tissue and inbred-specific regulation of gene expression

The active marks studied here appeared to reflect transcription levels (Fig. 1a). To investigate this correlation, expressed genes were binned into 5 quintiles based on their expression levels (RPKM) to compare with enrichment levels of active histone marks (Supplementary Fig. 4a). As expected from previous observations, a positive correlation could be seen, with highly expressed genes showing higher H3K27ac and H3K4me3 enrichment at the TSS, and higher H3K4me1 in the gene body^10,24,26^. Interestingly, the differences between the Top 20% and the 20–40% groups were not seen for H3K4me1, suggesting that a threshold was reached for this mark. Further confirming that these marks were associated with active genes in a tissue-specific manner, H3K27ac from immature ears was higher at genes up-regulated in ears versus all other tissues than at genes down-regulated in ears versus all other tissues (Supplementary Fig. 4b). Conversely, the same genes showed the opposite pattern in other tissues, such as in roots where H3K27ac was higher in genes down-regulated in ears compared to other tissues (Supplementary Fig. 4b). Similar trends were observed for H3K4me3, notably at the TSS, however H3K4me1 did not follow this trend. The same set of genes had higher H3K4me1 levels in both tissues, whether up-regulated in that tissue or not, suggesting that H3K4me1 was less variable across tissues and potentially not only correlated with gene expression^54^.

In addition to tissue-specific expression, our data enabled comparison of tissue-specific expression between the different inbreds. *BOOSTER 1 (B1)* is a regulator of anthocyanin metabolism, and the *B1-I* allele engages in paramutation^55^. In addition to the hepta-repeat that is responsible for paramutation in *B1-I*, another tissue-specific enhancer is present about 45 kb upstream from the TSS of the gene in B73^56^. This region was indeed marked by a H3K27ac peak in the coleoptilar nodes of B73 but not in the immature ears, correlating with *B1* expression and coleoptile pigmentation (Supplementary Fig. 5a). In W22, the enhancer was slightly closer (~20 kb) to the transcription start site, due to structural variation caused by TEs, and the enhancer was active in immature ears, correlating with gene expression (Supplementary Fig. 5b). This may be related to pigmentation of W22 under the control of *B1-bar*^57^.

### Identification of enhancers with bi-directional enhancer RNAs

**Fig. 5:**
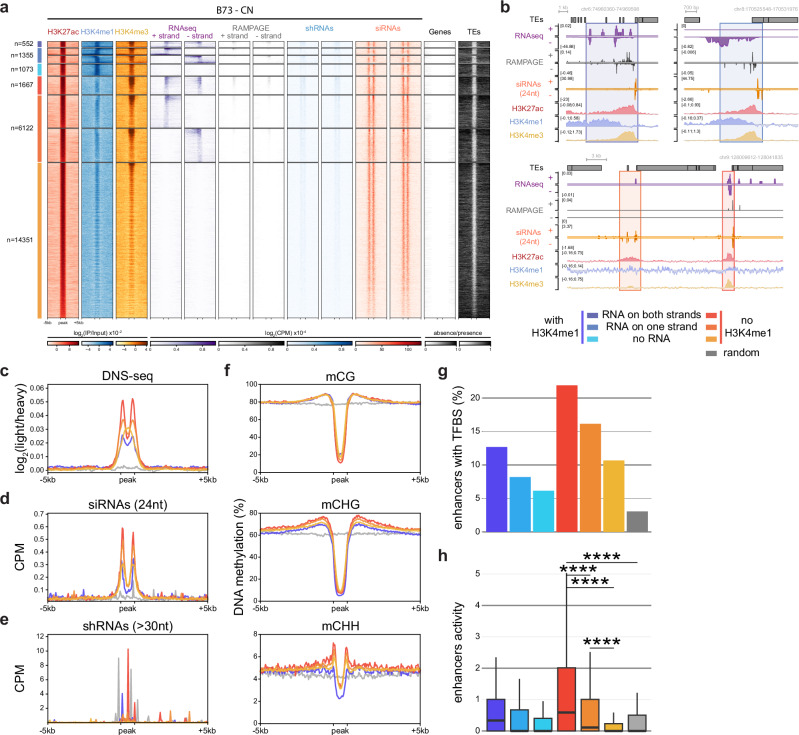
Enhancers with bi-directional enhancer RNAs are associated with stronger activity and higher RdDM at their boundaries. **a** Heatmap of ChIP-seq and transcriptomic signals in B73 coleoptilar node (CN) at distal H3K27ac peaks and ±5kb surrounding regions. Six classes of regulatory regions were identified based on the presence (blue) or the absence (red) of H3K4me1 peaks within 1 kb, and on the presence of RNA-seq reads mapping to both strands, one strand, or none (from darker to lighter shades). The short RNA-seq datasets were split into longer fragments (>30nt) and canonical siRNAs (24 nt). Presence (black) and absence (white) of annotated genes and TEs surrounding the peaks are shown, demonstrating the absence of annotated features within regulatory regions. **b** Browser screenshots of representative examples of uni- and bi-directionally expressed H3K27ac peaks (boxed), with (upper) and without (lower) H3K4me1 peaks. H3K4me1 peaks indicate the presence of unannotated genes. **c–f** Metaplots at the three clusters without H3K4me1 peaks (red, as in **a**), the three clusters with H3K4me1 peaks merged together (blue), and random control regions (gray) of DNA accessibility (**c**) in differential nucleosome sensitivity (DNS-seq) from CN^35^, 24nt siRNAs (**d**) and short RNAs (>30nt) (**e**) generated in CN in this study, as well as DNA methylation in each sequence context (**f**) from seedlings^59^. These metaplots show that the bi-directional enhancers are more accessible regions with higher transcription levels of shRNAs, depleted of DNA methylation, but also more protected from neighboring TEs by targeting of RNA-directed DNA methylation by 24nt siRNAs. **g** Percentage of peaks containing at least one transcription factor binding site (TFBS) from the TFs analyzed in this study. **h** Measure of enhancer activity for each cluster by STARR-seq^10^. Bi-directionally expressed enhancers drive statistically higher transcription (STARR-seq value within the enhancer) than uni-directional, not expressed or control regions (two-sided *t* test, **** p < 10^−5^). Data shows distribution of median STARR-seq value at all B73 CN enhancers (numbers shown in **a**), with the boxplot showing the mean and ranging from first to third quartiles, whiskers mark 1.5×IQR, and outliers are not shown.

When focusing on the distal regulatory regions—as defined by H3K27ac peaks at least 2 kb away from the closest annotated gene - we noticed that these regions with higher H3K27ac levels also had RNA-seq and RAMPAGE signals. We expected some of these signatures to be caused by mis-annotations, either an unannotated gene or a wrongly annotated TSS. Since we noted that gene bodies were marked by H3K4me1^26^, but that distal OCRs defined by ATAC-seq were depleted of H3K4me1 (Fig. 1a, b), we intersected H3K27ac peaks with H3K4me1 peaks in order to differentiate misannotated genes from bona fide enhancers. We allowed the H3K4me1 peak to be within 1 kb of the enhancers, to account for the distance between the TSS and the gene body (Fig. 5a; Supplementary Fig. 6a). As expected, loci with both histone marks had the same molecular characteristics as genes (Fig. 5b; Supplementary Fig. 6b), and were more often and more highly expressed in the corresponding tissue (Fig. 5a; Supplementary Fig. 6a–c), thus likely representing misannotated genes.

Interestingly, many distal H3K27ac peaks without H3K4me1 were also transcribed, either on one strand or on both strands (Fig. 5a, b; Supplementary Fig. 6a, b). These bi-directionally expressed non-coding RNAs had additional molecular signatures reminiscent of animal enhancer RNAs (eRNAs), notably the presence of a 5’-cap, as shown by RAMPAGE (Fig. 5a, b; Supplementary Fig. 6a, b). In addition, enhancers with bi-directional eRNAs had higher levels of H3K27ac and H3K4me3 (Supplementary Fig. 6c), and were more differentially accessible to MNase (Fig. 5c)^35^. In maize, RdDM targets mCHH islands neighboring genes and *cis*-regulatory elements^58,59^. Consistently, we observed high levels of 24nt siRNAs at the borders of enhancers, especially those with bi-directional eRNAs (Fig. 5d; Supplementary Fig. 6c), which accompany higher DNA methylation (Fig. 5f) in seedlings^59^. On the other hand, shRNAs (30–150 nt), including presumptive Pol V transcripts, were mostly produced within the enhancer region (Fig. 5e; Supplementary Fig. 6c).

**Fig. 6:**
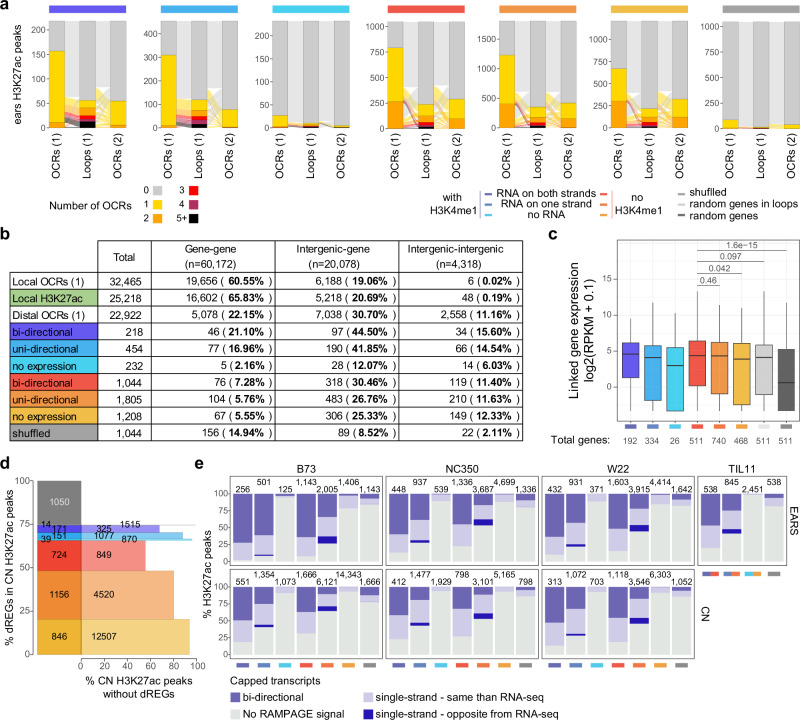
Enhancer RNA-expressing regions are enriched in chromatin loops. **a** Alluvial plots showing the number of open chromatin regions (OCRs) intersecting H3K27ac peaks, split by the presence of H3K4me1 peak within 1 kb, and the presence of RNA within the peaks. H3K27ac peaks identified in B73 immature ears were compared to OCRs from ATAC-seq and to chromatin loops from Hi-C from (1) Sun et al. ^38^ and to OCRs from (2) Ricci et al. ^10^. The highest overlap is between Sun et al. ^38^ OCRs and enhancers with bi-directional enhancer RNAs (eRNAs). **b** Table summarizing the number of enhancers found in the chromatin loop anchors identified by Hi-C^38^. H3K27ac peaks within 2 kb of a gene body (local H3K27ac, green) are more often in a loop than local OCRs. Distal H3K27ac peaks are included in intergenic loops to similar levels than OCRs. The presence of H3K4me1 however increases the percentage of these regions to be within loops, which support their classification as misannotated genes. **c** Expression level in immature ears (log_2_(RPKM + 0.1)) of the genes linked by chromatin loops to the different types of enhancers described in (**a**). Genes linked to enhancers with bi-directional eRNAs are more highly expressed than random genes, but marginally more highly expressed than random genes in loops (two-sided *t* test). **d** Intersection between elements with bi-directional nascent transcripts identified by discriminative regulatory-element (dREGs) in maize GRO-seq data^33^ and H3K27ac peaks in the coleoptilar node (CN). **e** Percentage of H3K27ac peaks with RAMPAGE signal, in immature ears and CN of each inbred. From 30 to 70% of enhancer RNAs are capped in bi-directional enhancers, while 10 to 30% of enhancers with stranded RNA-seq transcripts also have bi-directional RAMPAGE signal, suggesting an underestimation of the total number of bi-directional enhancers.

Validating the importance of these enhancers in gene regulation, TFBS were more often in regions of bi-directional eRNAs than in control regions of similar sizes (“Methods”), or than in the misannotated genes with H3K4me1 (Fig. 5g). Furthermore, by comparing to previously published STARR-seq data^10^, we found that enhancers with bi-directional eRNAs had higher enhancer activity than other distal enhancers or control regions (Fig. 5h). Despite their strong enhancer activity in vitro, these enhancers appeared to be expressed mostly in a tissue-specific manner in vivo (Supplementary Fig. 6d). These enhancers were also longer than enhancers without transcripts, but limited in size to several kilobases in all maize tissues (Supplementary Fig. 7a), shorter than “super-enhancers” in mammals which average several tens of kilobases^21^. The enhancer length did not seem to influence enhancer activity, since activity was higher in enhancers with bi-directional eRNAs in all tissues, whether measured by the maximum, the mean, or the median STARR-seq value in each enhancer (Supplementary Fig. 7b). Further supporting the activity of these enhancers, enhancers with bi-directional eRNAs showed the highest overlap with OCRs identified by ATAC-seq in comparable immature ears from other studies^10,38^, often including 2 OCRs within the same enhancer (Fig. 6a).

We then attempted to link these enhancers to the genes they regulate by intersecting putative enhancer regions with chromatin loops previously identified by chromatin conformation capture (Hi-C)^38^. Around half of the enhancers with bi-directional eRNAs were present in chromatin loops, in similar proportions as previously identified distal OCRs (Fig. 6b). Conversely, misannotated genes, marked by H3K4me1, were more often at chromatin loop anchors (Fig. 6b), which are enriched in gene-gene contacts. By comparison, local H3K27ac peaks were highly represented in gene-gene chromatin loops, slightly more (65% vs 60%) than local OCRs identified by ATAC-seq. Genes linked to enhancers with bi-directional eRNAs were more highly expressed than randomly selected genes, but only marginally more highly expressed than random genes present in chromatin loops (Fig. 6c), which already represent a subset of highly expressed genes.

The existence of enhancer RNAs in plants has been controversial, although recently bi-directional transcripts were identified at some regulatory regions in Arabidopsis^60^. In maize, analysis of nascent transcripts by GRO-seq initially failed to detect bi-directional transcripts at distal non-coding regions^61^, but subsequent reanalysis of the same data using the discriminative regulatory-element detection (dREG) algorithm^62^ identified around 4000 such regions in seedling shoots^33^. These regions corresponded to around half of the bi-directionally transcribed enhancers detected by MaizeCODE in the CN (Fig. 6d), as well as to some misannotated genes. RAMPAGE data indicated that 30 to 70% of the transcripts identified at the distal enhancers were capped, with similar levels in all tissues and inbreds analyzed (Fig. 6e). In addition, about 30% of H3K27ac peaks without H3K4me1 and with single-stranded RNA-seq expression also showed bi-directional GRO-seq and/or RAMPAGE signal (Fig. 6e), suggesting that the total number of enhancers with bi-directional eRNAs is underestimated in our datasets.

### Evolution of gene regulation during evolution and domestication

**Fig. 7:**
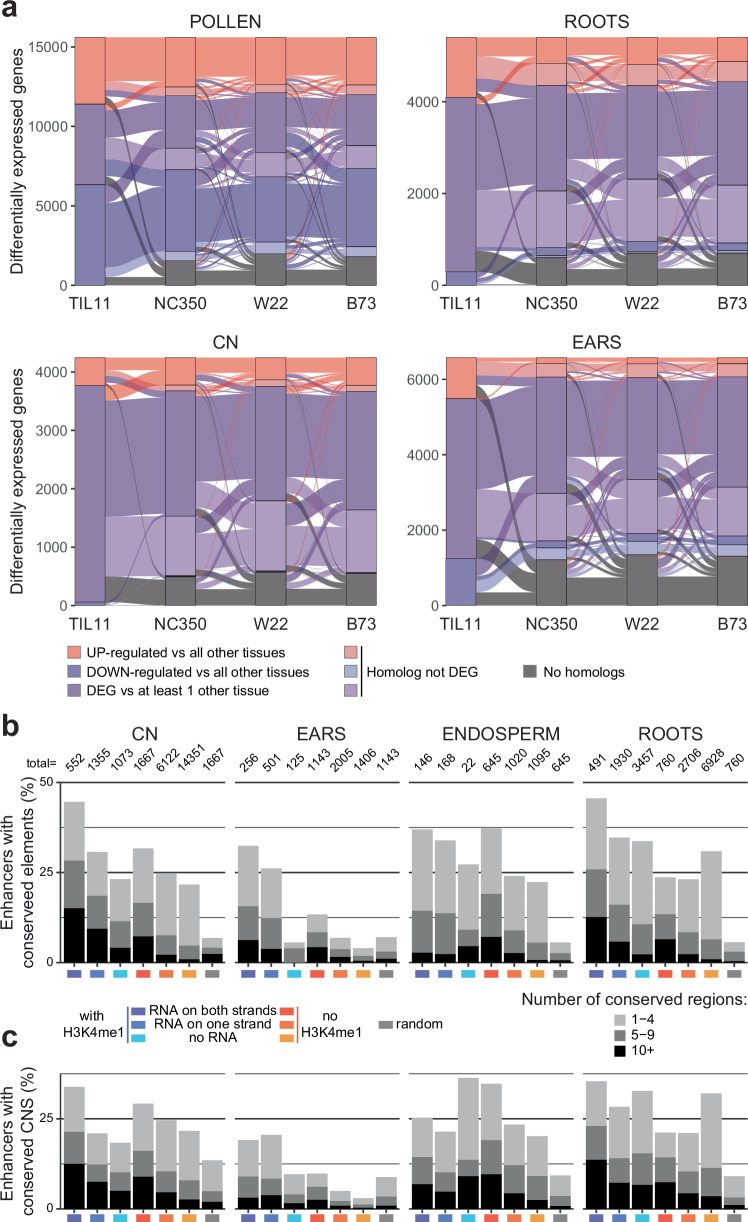
Domestication had a greater impact on transcription profiles and enhancersin ears. **a** Alluvial plot showing the differentially expressed genes (DEGs) in four tissues of TIL11, and whether their homologs in modern maize maintain this differential expression. These plots show high level of transcription profile conservation in pollen, moderate levels in coleoptilar nodes (CN) and root tips, and low levels in immature ears, in addition to more genes not having a homolog (“Methods”). **b** Percentage of enhancers containing conserved regions in the pan-andropoganeae clade identified by PhastCons (“Methods”). **c** Percentage of enhancers containing conserved regions identified by Conservatory CNS (“Methods”). In both conservation analyses, misannotated genes show high levels of conservation as do the enhancers, especially the ones with bi-directional enhancer RNAs, in all tissues but in immature ears.

To investigate the impact of domestication on differential transcription, we set about comparing gene regulation between TIL11 and modern maize inbreds. Genes that were differentially expressed in each TIL11 tissue were compared to their closest homologs in other inbreds (“Methods”) to ask if they were also differentially expressed (Fig. 7a). As noted earlier, pollen had the most differentially expressed genes, and this pattern was observed in all inbreds including TIL11 (Fig. 3; Supplementary Fig. 3). From the 10,531 DEGs in TIL11 pollen versus all other tissues, almost two thirds of their homologs were also DEGs in NC350 and B73 pollen versus all other tissues (65%, 62% respectively), and 57% were also DEGs in W22 pollen versus all other tissues (Fig. 7a). In coleoptilar nodes and in root tips, these proportions were slightly reduced, between 30 and 40% in the three inbreds. This proportion was drastically decreased in immature ears, where 10% or less of the genes differentially expressed in teosinte retained tissue-specificity in modern maize inbreds, including many novel genes in maize with no close homolog in teosinte (Fig. 7a; “Methods”). Overall, these results demonstrate that among the tissues studied here, tissue-specific gene expression evolved most rapidly in immature ears.

We next performed H3K27ac ChIP-seq in immature ears of TIL11, and identified distal putative regulatory regions with similar molecular signatures as in the modern maize inbreds (Supplementary Table 1; Supplementary Fig. 2a; Fig. 6e). We found similar small RNA signatures in TIL11 as in modern inbreds, with 24nt siRNAs targeting RdDM at the boundaries of active distal regulatory regions, while shRNAs were expressed within the regulatory regions (Supplementary Fig. 8). We next assessed the impact of domestication on tissue-specific *cis*-regulation by intersecting the different clusters of distal H3K27ac peaks identified above with conserved regions defined using PhastCons^63^ on the whole pan-andropogoneae clade (“Methods”). Between 25% and 50% of distal H3K27ac peaks neighboring H3K4me1 peaks had at least one—and often more than 10 - conserved regions, in all tissues, consistent with misannotated genes (Fig. 7b). In pollen, CN and roots, the remaining distal enhancers (those without H3K4me1) also had a higher number of conserved regions, correlating with an increase in eRNA transcription (Fig. 7b). However, in ears, a much lower number of enhancers contained conserved regions, barely higher than the control regions (Fig. 7b). These results indicate that *cis*-regulatory elements driving tissue-specific expression in maize ears were impacted by domestication. To further examine the conservation of these enhancers, we used the conserved non-coding sequences (CNS) identified by the Conservatory Project^64^. Higher numbers of conserved regions were again found in enhancers with bi-directional eRNAs from all tissues, except from immature ears (Fig. 7c). This analysis supports the idea that these “super-enhancers” have been conserved throughout the Poaceae, but that the ones driving differential expression in the ears of modern maize are not conserved.

## Discussion

The MaizeCODE initiative follows in the footsteps of the ENCODE project^17,18,65^ in cataloging regulatory regions in different tissues and inbred lines, so as to better understand the diversity of transcriptional regulation in maize. Additional datasets from teosinte enabled the analysis of tissue-specific transcription regulation in the context of domestication. In each inbred, most active histone marks were shared between all tissues tested (Fig. 1d). B73 immature ears had around 33,000 H3K27ac peaks in total (Fig. 1c), whereas more than 25,000 H3K27ac peaks were distal in coleoptilar nodes (Fig. 5a). It is likely that variation in the number of active enhancers in each tissue is caused by the heterogeneous composition of cell-types, a prediction borne out by single cell ATAC-seq studies of open chromatin regions (OCRs) in similar tissues^28^. Importantly, up to one quarter of the enhancers identified here in 5–10 mm immature ears by H3K27ac did not overlap with OCRs from 2 to 5 mm immature ears^38^, highlighting the dynamic regulation of histone modifications and chromatin accessibility during plant development. In W22, however, immature ears and coleoptilar nodes had about 40,000 H3K27ac peaks, among which about 2,500 peaks were specific to each tissue, and about 2,500 were shared only between these two tissues (Fig. 1d). Despite such large variation, the molecular signatures at enhancers, and notably the presence of capped, poly-A tailed, bi-directional enhancer RNAs were identified in all inbreds and tissues studied here (Fig. 5; Fig. 6e; Supplementary Fig. 6). The overall number of such “super-enhancers” was less variable between tissues (Supplementary Fig. 6d), although we cannot exclude the possibility that enhancers more highly enriched in H3K27ac (Supplementary Fig. 6c) were the easiest to identify. It is important to note that variation in the quality of chromatin preparations inherent to the different tissues, with additional contributions from sequencing depth and the peak calling algorithm could have a large impact on the number of peaks, and thus enhancers, identified (see Supplementary Data 2 for sequencing metrics). Nonetheless, biological variation is very significant. For example, *BOOSTER 1* (*B1*) has a conserved regulatory region which is active in coleoptilar nodes in B73, but drives expression in immature ears in W22 (Supplementary Fig. 5). *B1* is responsible for coleoptile pigmentation in B73, and glume pigmentation in tassels of W22. Differences in TE insertions in the region between the enhancer and the TSS in B73 and W22 could be responsible for this effect. Linking distal enhancers to their target genes can be done using chromatin conformation capture (Hi-C)^38,66^. We found that our enhancers were as often included in chromatin loops as OCRs marked by ATAC-seq (Fig. 6b), yet the expression level of the genes they are contacting were only marginally increased compared to random genes forming loops (Fig. 6c). Further studies would be required to more precisely associate enhancers to the genes they regulate, and to allow comparison between the different tissues and inbreds.

While the existence of transcripts at distal enhancers in plants has now been shown in multiple species through RNA-seq, capped small RNA-seq or global run-on sequencing^27,33,38,60^, their functions are less clear than in mammalian systems^22,27^. The number of distal H3K27ac peaks with bi-directional transcripts found in our data is consistent with these studies (~10% depending on the tissue, Fig. 5a; Supplementary Fig. 6a). These bi-directional transcripts were identified by RNA-seq amplified using poly(dT) and random primers, and not all were capped (Fig. 6), thus likely including both stable and unstable transcripts. Together, our results and that of other studies overwhelming support the existence of bi-directional eRNAs in plants, and their important role during development. Considering the differences in genome organization and transcription regulation between plant and mammalian systems^22,67–70^, it is however likely that they serve a different molecular function in plants, which constitute an exciting prospect in the field.

In mammalian genomes, H3K4me1 is associated with enhancers, whether poised or active^19,39^. In plants, H3K4me1 is associated with gene bodies^71,72^, and its pattern in all maize tissues and inbreds was consistent with previous studies^10^ (Fig. 1). In this study, we went one step further and used H3K4me1 as a proxy for genes (Fig. 5; Supplementary Fig. 6). Validating this approach, distal H3K27ac peaks marked by H3K4me1 were more often present at chromatin loop anchors than bona fide enhancers, or than OCRs identified with ATAC-seq^38^ (Fig. 6). This is consistent with previous work in maize that found that unmethylated regions of the genome (UMRs) with inaccessible chromatin had higher H3K4me1 levels than accessible ones, unlike H3K27ac and H3K4me3, which were higher in accessible UMRs^59^. H3K4me1 can be deposited in transcription-dependent and independent mechanisms^54^, potentially explaining the seemingly contradictory results of H3K4me1 being positively correlated with transcription, yet not being correlated with differential expression (Supplementary Fig. 4). H4K16ac (as well as H2B ubiquitination) has been implicated in the recruitment of H3K4me1 methyltransferase^54^, and prevents chromatin remodeling by the epigenetic regulator DDM1^73^, which is found at RdDM targets in maize^74,75^. These observations suggest that H3K4me1 is present on genes that are not being silenced by DDM1, potentially allowing transcription elongation or preventing ectopic RdDM.

Transposable elements are drivers of *cis*-regulatory elements in plant genomes^76^, and the regulation of *tb1* is a well-known example of their impact on maize domestication^10,77^. TEs are under tight epigenetic control during the life-cycle of plants, and mechanisms responsible for keeping them in check include small RNAs and RdDM^78–80^. In maize, epigenetic signatures of mCHH methylation and 24nt siRNAs are found at gene boundaries, presumably to prevent euchromatic marks leaking into silenced TEs^58,59,81^. We also found that siRNAs and mCHH methylation are sharply elevated at the borders of distal regulatory elements in both modern maize and in teosinte (Fig. 4; Supplementary Figs. 6, 8). In addition, levels of siRNA, shRNA and mCHH were positively correlated with enhancer strength, being higher in enhancers with bi-directional eRNAs, than enhancers with eRNAs on one strand, than enhancers without eRNA (Fig. 5a, d; Supplementary Fig. 6a, c). A possible explanation for this observation is that enhancer strength and increased DNA accessibility (Fig. 5; Supplementary Fig. 6) enable easier access to RNA polymerase IV and Pol V (and therefore to the RdDM machinery) in the same way as they enable access to Pol II (Supplementary Fig. 9). It is thus possible that the role of RdDM in maize – to prevent leakage of active transcription from enhancers into surrounding TEs – originates from the expression of enhancer RNAs.

In pollen, as previously observed^47^, TEs are strongly upregulated, with almost half of all unique RAMPAGE signals overlapping with TE annotation (Fig. 3c). Similar results were obtained in endosperm, which shared other features with pollen including differential expression of telomere maintenance genes (Fig. 3b; Supplementary Fig. 3b, c). Intriguingly, small RNA sequencing revealed pollen-specific clusters of small RNAs on each chromosome, derived from long non-coding hairpins (Fig. 4). Overlapping 21, 22, and 24nt hairpin small RNAs from the same sequence in otherwise very long hairpins, are a feature of proto-miRNAs, thought to be the precursors of miRNAs in plants^82^. In teosinte *mexicana*, one such cluster on chromosome 5 has recently been identified as a selfish genetic element responsible for *Teosinte Pollen Drive*, a gamete-killing incompatibility in teosinte hybrids with maize inbred W22^52^. Variation among pollen specific proto-miRNA in modern maize reported here could reflect a history of similar hybridizations during domestication and diversification.

Consistent with the focus of breeding and domestication on yield and harvest traits, transcriptional regulation in immature maize ears showed very little conservation with teosinte, both in terms of patterns of expression of orthologous genes (Fig. 7a) and of their *cis*-regulatory elements (Fig. 7b, c). These results suggest that enhancers were not only reshuffled by TE insertions, as in the case of *tb1*, but evolved as rapidly as the genes they regulate, while maintaining their ability to drive strong transcription during domestication (Fig. 5e; Supplementary Fig. 7b). The highest level of transcriptional conservation between maize and teosinte was found in pollen (Fig. 7a), despite having the most unique transcriptional profile of all the tissues examined for both coding and non-coding RNAs (Fig. 3; Fig. 4; Supplementary Fig. 3). This transcriptional profile is likely representative of conserved functions in reproduction, since breeding relies on fecundity and genome stability. It is also possible that conservation of pollen gene expression between maize and teosinte, which is otherwise unique among tissues, is the result of gene drive mechanisms such as *Teosinte Pollen Drive*^52^, that could be responsible for the fixation of epigenetic factors in modern maize varieties, and for the establishment of the molecular signatures identified here at their regulatory regions.

## Methods

### Plant material and growth conditions

Seeds stocks for B73, W22 and NC350 were obtained from the Maize Genetics Stock Center, and for TIL11 from Dr. John Doebley.

For collecting immature ears, maize inbreds were grown in the CSHL Uplands Farm field in the summer until they reached the appropriate stage. The plants were collected from the field, and 5–10 mm primary and secondary ear primordia were dissected in the lab then frozen in LN_2_ and stored at –80 °C. TIL11 plants were grown in CSHL Upland Farm field from September to early October to promote floral transition by natural short day conditions. Immature TIL11 ears at an equivalent development stage (with inflorescence meristems, spikelet pair meristems, spikelet meristems and floral meristems) were collected under a dissecting microscope, frozen in LN_2_ and stored at –80 °C.

For maize pollen samples, shedding tassels of field-grown plants as described above were bagged in the evening and mature pollen was collected the following day. After passing through a sieve to remove anthers, pollen was frozen in LN_2_ and stored at –80 °C.

For harvesting TIL11 pollen, plants were grown in a short day (8 h light/16 h dark) walk-in chamber to promote floral transition. Fresh pollen was harvested, frozen in LN_2_ and stored at –80 °C.

For maize endosperm samples, ears of field-grown plants were sib-pollinated and collected 15 DAP. Endosperm was dissected in the lab, frozen in LN_2_ and stored in –80 °C.

For maize and teosinte root tip samples, seeds were germinated on wet paper towels in a Pyrex dish in an incubator at 26 °C in continuous darkness. After 5 days, 1–3 mm root tips were cut off with a razor blade on ice, frozen in LN_2_ and stored at –80 °C.

For maize and teosinte coleoptilar nodes samples, seeds were germinated in flats in a long day (8 h dark/16 h light) growth chamber, 27 °C day and 24 °C night, and light at 130 μmoles. After 5 days, seedlings were unearthed and 5 mm sections around coleoptilar nodes were dissected on ice, frozen in LN_2_ and stored at –80 °C.

At least three biological replicates of each tissue were collected.

### PacBio HiFi and ONT long-reads whole-genome sequencing of TIL11

Extracted DNA from TIL11 leaf nuclei was analyzed by Femto Pulse to assess fragment length distribution. For PacBio HiFi, DNA was sheared to ∼15 kb using a Diagnode Megarupter following manufacturer’s recommendations. DNA was prepared for PacBio sequencing using the PacBio template prep kit 10. Briefly, 5ug of fragmented DNA prepared for sequencing via the PacBio kit, prepared libraries were size selected on Blue Pippin (Sage) from 10–15 kb, and sequencing primer v2 was used. The library was loaded at 70pM on a PacBio Sequel II with a 48 h movie. Circular Consensus processing was performed in SMRTLink to ensure multiple passes per fragment, and >=Q20 reads were selected for downstream assembly.

For ONT long reads sequencing, DNA was sheared to ∼30 kb using a Diagnode Megarupter following manufacturer’s recommendations. DNA was prepared for Nanopore sequencing using the ONT 1D sequencing by ligation kit (SQK-LSK109). Briefly, 2 μg of fragmented DNA was repaired with the NEB FFPE repair kit, followed by end repair and A-tailing with the NEB Ultra II end-prep kit. After an Ampure clean-up step, prepared fragments were ligated to ONT specific adapters via the NEB blunt/TA master mix kit. The library underwent a final clean-up and was loaded onto a PromethION PRO0002 flow cell per manufacturer’s instructions. The flowcells were sequenced with standard parameters for 3 days. Basecalling was performed with Guppy V5 to increase quality.

### Optical map generation

BioNano Optical Mapping was performed at Corteva Agriscience (Indianapolis, IN) following the protocols optimized for the NAM genomes^31^. Briefly, high molecular weight DNA was collected from fresh tissue from seedlings using the Bionano Prep™ Plant Tissue DNA Isolation Kit. Labeling was performed using the DLS Kit (Bionano Genomics Cat.80005) following manufacturer’s recommendations along with optimizations from the NAM samples. DNA was stained and quantified by adding Bionano DNA Stain to a final concentration of 1 microliter per 0.1 microgram of DNA. The labeled sample was then loaded onto a Bionano chip flow cell where molecules were separated, imaged, and digitized in the Saphyr System according to the manufacturer’s recommendations (https://bionanogenomics.com/support-page/saphyr-system/). Data visualization, processing, and DLS map assembly were conducted using the Bionano Genomics software Access, Solve and Tools.

### TIL11 genome assembly and assessment

Prior to the genome assembly, we first assessed the size and heterozygosity of the TIL11 genome by analyzing the frequency distribution of 21-mers within the PacBio HiFi reads using KMC v3.1.1^83^ and GenomeScope v2.0^84^. This analysis confirmed the high quality of the HiFi reads and very low rates of residual heterozygosity (<0.001%) with on average 22x coverage in reads averaging 11.7kbp. Following this initial evaluation, we proceeded with the de novo assembly of long reads from PacBio HiFi Sequencing data using HiCanu^84,85^ optimized for high-fidelity long reads. The resultant assembly spanned 2.397 Gb with a contig N50 of 22.4Mbp (max: 95.0Mbp). These contigs were then scaffolded and packaged following the protocol used for the Maize NAM accessions^31^ using BioNano optical mapping data with the Bionano Access software and ALLMAPS. This yielded a highly contiguous & accurate, chromosome scale assembly with a scaffold N50 of 229.43Mbp and a contig N50 of 45.03Mbp.

We assessed both consensus accuracy and completeness by analyzing the HiFi k-mer copy number spectra using Merqury version 2020-01-29^86^. Additionally, to gauge assembly completeness, we employed BUSCO v5.0.0^86,87^ with the embryophyta database from OrthoDBv10^88^ in genome mode. We investigated augmenting the assembly using the ONT long reads but found only potentially marginal improvements so did not include these results. Assembly based Structural Variants (SV) were characterized by aligning the chromosome scale assemblies of B73v5, NC350 and W22 lines to TIL11 using winnomap^89^ and further analyzing them using the SyRI package^90^.

### TIL11 annotations and gene orthology

Gene annotations for the TIL11 genome were done using the same protocol as described for the NAM genomes^31^. Orthologous genes were called using the Ensembl Compara Trees^91^. We dumped orthologs between two species from ensembl compara database with API. The orthology is a subclass of homology in the compara database. It was assigned by compara pipeline after reconciliation between gene tree and species tree. For any pair of homologs in a gene family, if their most recent common ancestor went through speciation event, these two homologs were deemed as orthologs. The annotation for TIL11 and comparative analysis with other NAM genomes is available on Gramene Maize (https://maize-pangenome.gramene.org/).

### Chromatin immuno-precipitation sequencing (ChIP-seq) of histone modifications

The following amounts of tissues were used for each chromatin preparation: 10 coleoptilar nodes, 150 root tips, 10 immature ears and 10 endosperms. Chromatin was extracted as previously described^92^. Briefly, tissue was fixed in PBS with 1% formaldehyde under vacuum for 30 min. Crosslinking was stopped by adding glycine solution to 0.1 M final concentration. Fixed tissue was ground with pestle and mortar in LN_2_ and further disrupted using a dounce homogenizer. Chromatin was sheared using Covaris ultrasonicator and 300 μl of the chromatin prep was used for each immunoprecipitation with exception of coleoptilar nodes where 500 μl was used. The following antibodies were used to target chromatin modifications: H3K4me1 (Abcam, ab8895), H3K4me3 (Millipore, 07-473) and H3K27ac (Abcam, ab4729). Mixture of Dynabeads with proteins A and G (1:1) (Invitrogen) was used to pull-down the protein/DNA complexes and DNA was purified using ChIP Clean-up and Concentrator kit (Zymo Research). Libraries were constructed using Ultra II DNA kit (NEB).

### ChIP-seq of transcription factors

TU1-A-YFP, the dominant duplication^12^, and GT1-YFP^11^ transgenic lines were introgressed into the *bd1;Tunicate* (*bd1;Tu*) double mutant background, which produces highly proliferative ears, to generate large amounts of ear tissue. ChIP experiments were adapted from a previously described protocol^44^. Briefly, two biological replicates of freshly harvested ear tissues were cross-linked in ice-cold buffer containing 10 mM HEPES-NaOH PH7.4, 1% formaldehyde, 0.4 M sucrose, 1 mM EDTA, and 1 mM PMSF, for 20 min under vacuum. Glycine was then added to a concentration of 0.1 M for another 5 min under vacuum to quench the crosslink. Nuclei extraction and immunoprecipitation were conducted as previously described^45^ using CelLytic PN Isolation/Extraction Kit (Sigma-Aldrich) and high-affinity GFP-Trap magnetic agarose (ChromoTek, gtma-20). ChIP-seq libraries were built as previously described^45^ using NEXTflex ChIP-seq Kit (PerkinElmer Applied Genomics) and AMPure XP beads (Beckman Coulter). ChIP-seq libraries were quantified by KAPA Library Quantification Kits (Roche) and sent for Illumina sequencing. ChIP-seq data generated from previous studies were used for ZmHDZIV6-YFP^45^, FEA4-YFP^44^, KN1^43^ and TB1^8^.

### Whole-transcriptome sequencing (RNA-seq)

For all inbreds and tissues, RNA was extracted with Direct-zol RNA Miniprep Kit (Zymo Research). 1 μg of total RNA was processed with the TruSeq Stranded Total RNA LT Kit (Illumina) as follows: all ribosomal RNAs were removed with RiboZero Plant included in the kit. After RNAclean XP purification (Beckman Coulter), anchored oligo(dT) and random probes were added and the RNA was fragmented. cDNA synthesis was performed followed by 2nd strand synthesis, 3’ adenylation, adapter ligation and target amplification. After purification with AMPure XP (Beckman Coulter) samples were quantified on a 2100 Bioanalyzer using a HS-DNA-Chip (Agilent), and adjusted to a concentration of 10 nM. Libraries were then pooled at equimolar concentration and sequenced on an Illumina NextSeq 500 Sequencer with a paired end 150 bp run.

### RNA annotation and mapping of promoters for the analysis of gene expression (RAMPAGE)

This protocol is a modified version of a previously published method^51^. Starting with 5 μg of total RNA, ribosomal RNAs were removed using the RiboMinus Plant Kit for RNA-Seq (Thermo Fisher Scientific) followed by incubation with Terminator 5′-Phosphate-Dependent Exonuclease (TEX) (Lucigen) to remove all residual RNAs containing 5’ monophosphate. We then performed first-strand synthesis using the SMARTer Stranded Total RNA Kit V2- Pico Input Mammalian (Takara). Following purification with RNAclean XP (Beckman Coulter), 5’ cap oxidation, 5’ cap biotinylation, RNase I digestion, and streptavidin pulldown (Cap Trapping) were performed as previously described^51^. Amplification of purified cDNAs (two rounds of PCR to attach Illumina adapters and amplify the libraries) followed by AMPure XP cleanup (Beckman Coulter) was done using the SMARTer Stranded Total RNA v2 kit (Takara), according to protocol. All samples were processed separately, quantitated on a 2100 Bioanalyzer using a HS-DNA-Chip (Agilent), and adjusted to a concentration of 10 nM. Libraries were then pooled at equimolar concentration and sequenced on an Illumina NextSeq 500 Sequencer.

### Total RNA short RNA sequencing (shRNA-seq)

5 μg of total RNA were first depleted of rRNA with the RiboMinus Plant Kit for RNA-Seq (Thermo Fisher Scientific). RNA was de-capped using Cap-Clip Pyrophosphatase. The Illumina Truseq Small RNA protocol was used as follows: 3’ and 5’ adapters were ligated, followed by reverse transcription and amplification of the library. The BluePippin Size Selection system (Sage Science) was used to select library fragments ranging from 100 to 205 nt with the 3% agarose gel cassettes. Samples were quantified on a 2100 Bioanalyzer using a HS-DNA-Chip (Agilent), and adjusted to a concentration of 5 nM. Libraries were then pooled at equimolar concentration and sequenced on an Illumina NextSeq 500 Sequencer using a single end 150 bp run.

### Data analysis pipeline

Data analysis was performed using the MaizeCODE pipeline and accompanying scripts: https://github.com/martienssenlab/maize-code.

In brief, adapters were trimmed from raw sequencing files with cutadapt^93^ and data quality was assessed before and after trimming with FastQC. For ChIP-seq, trimmed files were mapped with bowtie2^94^ and processed with samtools^95^.

Peaks were called with Macs2^96^ and transcription factor motifs with the Meme suite^97^. For all samples, the two biological replicates were merged after mapping, split randomly into two pseudo-replicates and only the peaks called in the merged sample as well as in both pseudo-replicates were selected. For TB1, using the peaks identified in both biological replicates by Irreproducible Discovery Rate (IDR)^98^ generated more accurate results.

For RNA and RAMPAGE, trimmed files were mapped with STAR^99^. Differential gene expression analysis for RNAseq was performed with EdgeR v3.32.1^100^ and Gene Ontology analysis with rrvgo v1.5.3^101^ and topGO v2.42.0^102^ from GO databases created with GOMAP^103^. Transcription start sites were called with Macs2 using RNAseq as controls. For shRNA-seq, trimmed files were depleted of structural RNAs by mapping to rRNAs, snoRNAs and tRNAs with bowtie2. The unmapped reads were then mapped with Shortstack^104^. Mapped reads of 20 to 24nt were used to call sRNA loci with Shortstack, whereas reads longer than 30nt were kept for shRNA tracks. For DNA methylation, published datasets were processed with Bismark^105^. Browser tracks for all types of data were generated with Deeptools^106^. Heatmaps and metaplots were also generated with Deeptools, except for gene expression heatmaps which were produced with gplots v3.1.3; Upset plots were generated with ComplexUpset v1.3.3^107^; browser shots with Gviz v1.34.1^108^; boxplots with ggplot2 v3.4.1^109^. The following R packages and their versions were also used for data processing and plotting: dplyr v1.1.0; tidyr v1.3.0; cowplot v1.1.1; RColorBrewer v1.1.3; AnnotationForge 1.32.0; purrr 1.0.1; limma 3.46.0; stringr 1.5.0; wesanderson 0.3.6.

See Supplementary Data 2 for all sequencing library metrics.

### Random control regions in mappable space

The B73 genome was fragmented into 150 bp non-overlapping bins, which were then treated as single-end reads and mapped back to their respective genome following the same pipeline as ChIP-seq datasets. Only regions of the genome with at least one read mapped were kept as mappable. Bi-directionally expressed enhancers from each tissue individually were then randomly shuffled within this mappable space using the bedtools shuffle command^110^ in order to keep the same number and size distribution.

### Analysis of conservation within enhancers

An Andropogoneae phylogeny was inferred based on all genome-wide fourfold degenerate sites with <50% Androgoneae-wide missingness using RAxML. A neutral model of evolution was fit to this phylogeny using phyloFit from the PHAST 1.4 package^63^. A set of most conserved elements was generated using the PhastCons “most-conserved” flag from the PHAST package with an expected length of 40 bp, after training to generate models of conserved and non-conserved elements using genome-wide multiple alignments with “--coverage 0.25”. To prevent reference-bias in the discovery of CNS, the B73 reference was masked and all other Tripsacineae were excluded for the phastCons analyses.

Conservatory CNSs were obtained from The Conservatory Project (www.conservatorycns.com)^64^.

### Statistics and reproducibility

No statistical method was used to predetermine sample size. No data were excluded from the analyses. The experiments were not randomized. The investigators were not blinded to allocation during experiments and outcome assessment.

### Reporting summary

Further information on research design is available in the Nature Portfolio Reporting Summary linked to this article.

## .Supplementary information

Supplementary Information

Peer Review File

Description of additional supplementary files

Supplementary Data 1

Supplementary Data 2

Reporting Summary

## Source data

Source Data

## Data Availability

The sequencing data generated in this study have been deposited in the Gene Expression Omnibus (GEO) database under accession code SuperSeries GSE254496. The processed data are available at https://maize-pangenome.gramene.org/. The ChIP-seq sequencing data used in this study are available in the GEO database under accession codes GSE61954 and GSE39161, or in the National Center for Biotechnology Information (NCBI) database under accession codes PRJNA517683 and PRJNA647198. Source data are provided with this paper.

## Code availability

All code is available on Github: 10.5281/zenodo.14275877^111^.
