# Author Correction: MaizeCODE reveals bi-directionally expressed enhancers that harbor molecular signatures of maize domestication

**DOI:** 10.1038/s41467-025-62179-x

**Published:** 2025-07-23

**Authors:** Jonathan Cahn, Michael Regulski, Jason Lynn, Evan Ernst, Cristiane de Santis Alves, Srividya Ramakrishnan, Kapeel Chougule, Sharon Wei, Zhenyuan Lu, Xiaosa Xu, Umamaheswari Ramu, Jorg Drenkow, Cassidy Danyko, Melissa Kramer, Arun Seetharam, Matthew B. Hufford, W. Richard McCombie, Doreen Ware, David Jackson, Michael C. Schatz, Thomas R. Gingeras, Robert A. Martienssen

**Affiliations:** 1 Howard Hughes Medical Institute, Cold Spring Harbor Laboratory, Cold Spring Harbor, NY USA; 2 Cold Spring Harbor Laboratory, Cold Spring Harbor, NY USA; 3 Johns Hopkins University, Baltimore, MD USA; 4 Department of Ecology, Evolution, and Organismal Biology, Iowa State University, Ames, IA USA; 5 USDA ARS Robert W. Holley Center for Agriculture and Health Cornell University, Ithaca, NY USA; 6 Present Address: Department of Plant Biology, University of California, Davis, CA USA

**Keywords:** Epigenomics, Histone post-translational modifications, Plant domestication, Transcriptional regulatory elements

Correction to: *Nature Communications*; 10.1038/s41467-024-55195-w, published online 30 December 2024

In the version of the article initially published, Cassidy Danyko (Cold Spring Harbor Laboratory, Cold Spring Harbor, NY, USA) did not appear in the author list or contributions (performed the experiments and sequencing), as is now amended in the HTML and PDF versions of the article.

