# Supplementary material for: MaizeCODE reveals bi-directionally expressed enhancers that harbor molecular signatures of maize domestication
Source: Nat Commun. 2024 Dec 30;15:10854. doi: 10.1038/s41467-024-55195-w (PMC11685423; doi:10.1038/s41467-024-55195-w)
Supplement: Supplementary file 1 — Supplementary Information [file 41467_2024_55195_MOESM1_ESM.pdf]

Supplementary Information

Supplementary Table 1. Datasets generated by the MaizeCODE consortium and analyzed in this study.

Supplementary Fig. 1. TIL11 assembly statistics and structural variation with modern maize inbreds.

Supplementary Fig. 2. Histone H3 modifications mark DNA regulatory elements in all tissues and all maize inbreds, and colocalize with open chromatin regions.

Supplementary Fig. 3. Similar tissue-specific transcriptional profiles in maize and teosinte inbreds.

Supplementary Fig. 4. Histone modification levels correlate with gene expression within tissues, but H3K4me1 does not correlate with differential expression between tissues.

Supplementary Fig. 5. Example of tissue-specific gene regulation in two inbreds: the *Booster1* (*B1*) locus.

Supplementary Fig. 6. Enhancers with bi-directional enhancer RNAs are tissue-specific.

Supplementary Fig. 7. Expressed enhancers are longer and inherently drive more expression, in all tissues.

Supplementary Fig. 8. Small interfering RNAs also target boundaries of enhancers in TIL11.

Supplementary Fig. 9. Model of enhancers with bi-directional enhancer RNAs.

Supplementary References

Supplementary Table 1. Datasets generated by the MaizeCODE consortium and analyzed in this study.
Previously published datasets are italicized and the publications listed in Supplementary References

		RNA			ChIP-seq			Accessiblity	TF ChIP-seq		
**Inbred**	**Tissue**	**RNAseq**	**RAMPAGE**	**shRNAseq**	**H3K27ac**	**H3K4me3**	**H3K4me1**	***DNS-seq*** [1]	**GT1**	**TU1A**	***HDZIV6*** [2]
B73	Immature ears	2 Reps	2 Reps	2 Reps	2 Reps	2 Reps	2 Reps	*2 Reps*	2 Reps	2 Reps	*2 Reps*
B73	Endosperm	2 Reps	2 Reps	2 Reps	2 Reps	2 Reps	2 Reps	*2 Reps*	-	-	-
B73	Root tips	2 Reps	2 Reps	2 Reps	2 Reps	2 Reps	2 Reps	*2 Reps*	-	-	-
B73	Coleoptilar nodes	2 Reps	2 Reps	2 Reps	2 Reps	2 Reps	2 Reps	*2 Reps*	-	-	-
B73	Pollen	2 Reps	2 Reps	2 Reps	-	-	-	-	-	-	-
W22	Immature ears	2 Reps	2 Reps	2 Reps	2 Reps	2 Reps	2 Reps	-	-	-	-
W22	Endosperm	2 Reps	2 Reps	2 Reps	2 Reps	2 Reps	2 Reps	-	-	-	-
W22	Root tips	2 Reps	2 Reps	2 Reps	2 Reps	2 Reps	2 Reps	-	-	-	-
W22	Coleoptilar nodes	2 Reps	2 Reps	2 Reps	2 Reps	2 Reps	2 Reps	-	-	-	-
W22	Pollen	2 Reps	2 Reps	2 Reps	-	-	-	-	-	-	-
NC350	Immature ears	2 Reps	2 Reps	2 Reps	2 Reps	2 Reps	2 Reps	-	-	-	-
NC350	Endosperm	2 Reps	2 Reps	2 Reps	2 Reps	2 Reps	2 Reps	-	-	-	-
NC350	Root tips	2 Reps	2 Reps	2 Reps	2 Reps	2 Reps	2 Reps	-	-	-	-
NC350	Coleoptilar nodes	2 Reps	2 Reps	2 Reps	2 Reps	2 Reps	2 Reps	-	-	-	-
NC350	Pollen	2 Reps	2 Reps	2 Reps	-	-	-	-	-	-	-
TIL11	Immature ears	2 Reps	2 Reps	2 Reps	2 Reps	-	-	-	-	-	-
TIL11	Coleoptilar nodes	2 Reps	2 Reps	2 Reps	-	-	-	-	-	-	-
TIL11	Pollen	2 Reps	2 Reps	2 Reps	-	-	-	-	-	-	-
TIL11	Root tips	2 Reps	-	-	-	-	-	-	-	-	-

a

Assembly Statistics TIL11 v1.1	
**Contigs**	
# contigs	9.803
Longest (Mbp)	109.55
N50 (Mbp)	45.03
**Scaffolds**	
# scaffolds	7,445
> 10Mbp	10
Span (Mbp)	2421
Gapped bases (%)	1.37
**Annotation**	
# genes	45,535
Interspersed repeats (%)	81.96
**Quality Control**	
Merqury QV estimate	66.9
Merqury completeness estimate	97.08
Complete BUSCOs	97.9
Missing BUSCOs	0.8

b

TIL11 Structural variation summary				
		**B73**	**NC350**	**W22**
**Variant Type**	**Size**			
**Sniffles**				
Deletion	200bp - 1kb	29,214	32,623	24,739
	1kb - 10kb	46,191	45,333	36,448
	> 10kb	55,635	56,278	48,959
Duplication	200bp - 1kb	578	553	1,052
	1kb - 10kb	2,563	2,760	3,491
	> 10kb	14,783	15,155	13,481
Inversion	200bp - 1kb	454	514	384
	1kb - 10kb	1,352	1,407	1,272
	> 10kb	28,365	28,942	73
Insertion	200bp - 1kb	22,183	25,078	21,061
	1kb - 10kb	12,066	12,501	10,412
	> 10kb	174	205	178
**Syri**				
Inversion	> 1Mbp	8	18	13

c

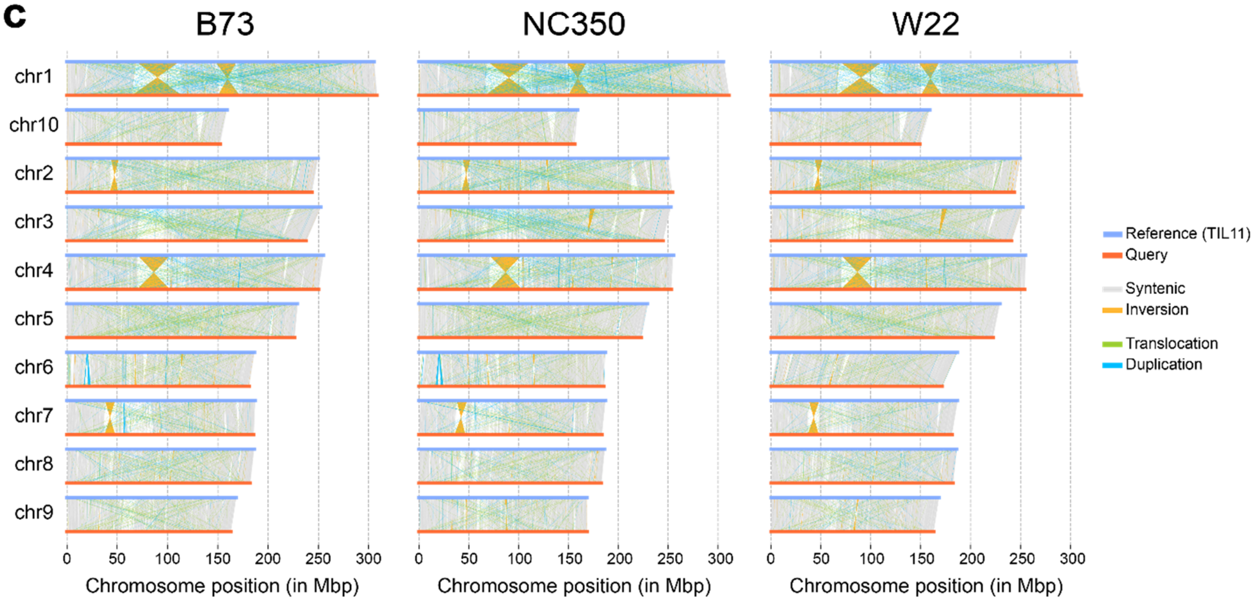

Supplementary Fig. 1. TIL11 assembly statistics and structural variation with modern maize inbreds.

a. Table of assembly and annotation statistics, showing high quality and comparable metrics with genomes assembled in the NAM project. **b.** Summary of the different structural variants between TIL11 and the three other inbreds studied. **c.** Full chromosome visualization of structural variants between TIL11 (used as reference) and the three other inbreds studied. Most large variants, and notably inversions, are conserved between maize inbreds.

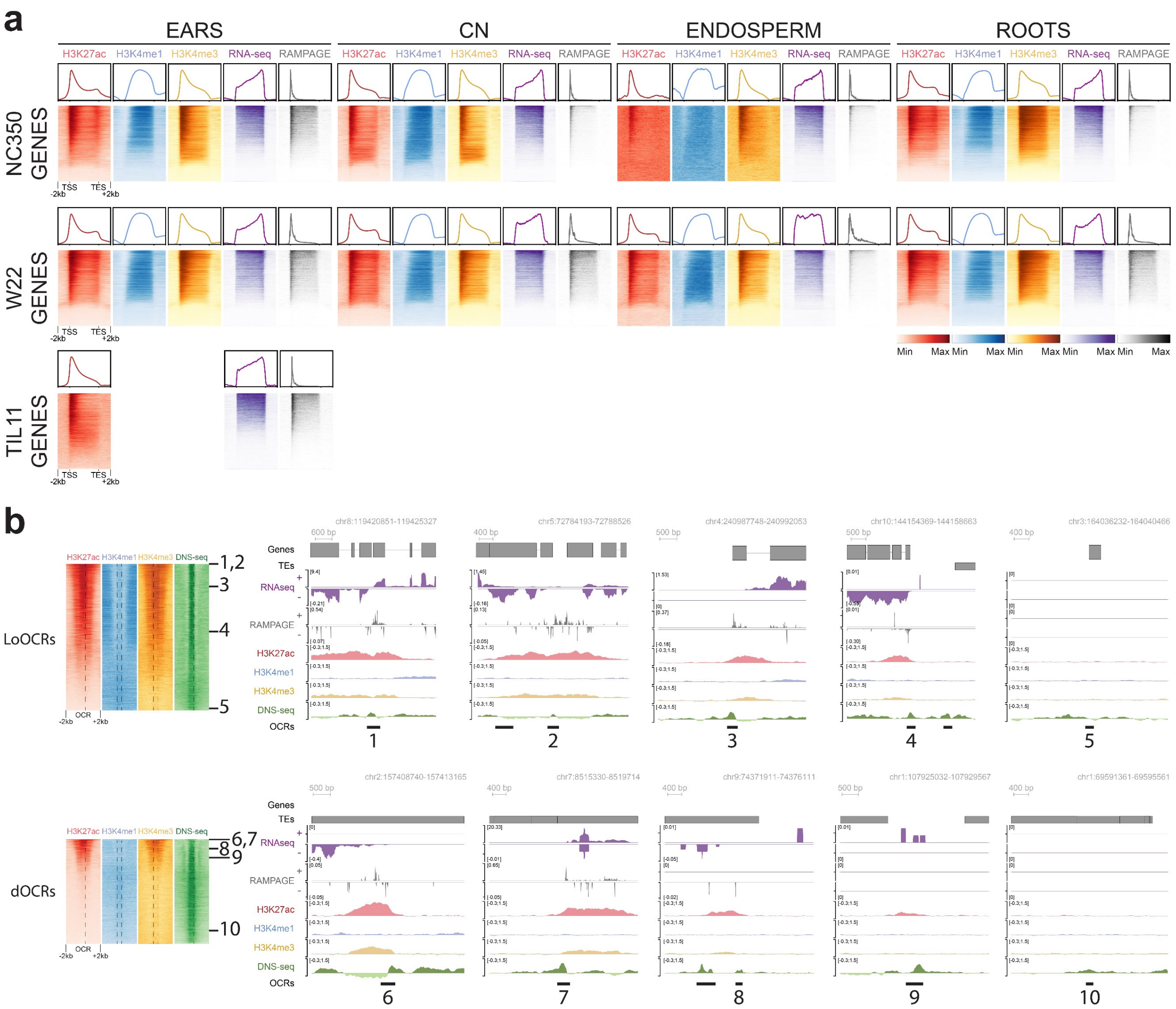

Supplementary Fig. 2. Histone H3 modifications mark DNA regulatory elements in all tissues and all maize inbreds, and colocalize with open chromatin regions.

a. Heatmaps and metaplots of H3K27ac, H3K4me1, H3K4me3, RNA-seq and RAMPAGE over all annotated genes in each tissue of each inbred (see **Fig. 1a** for B73). CN = coleoptilar node. **b.** Browser screenshots of example regions centered around local or distal open chromatin regions (LoOCR, dOCRs, respectively) previously identified by ATAC-seq[3]. RNA-seq (count per million, CPM), RAMPAGE (CPM), ChIP-seq (log_2[IP/Input]) and differential nucleosome sensitivity (DNS-seq, log_2[light/heavy digest]) are shown at each OCR numbered 1 to 10 on the left hand-side heatmap (same heatmap than **Fig. 1b**).

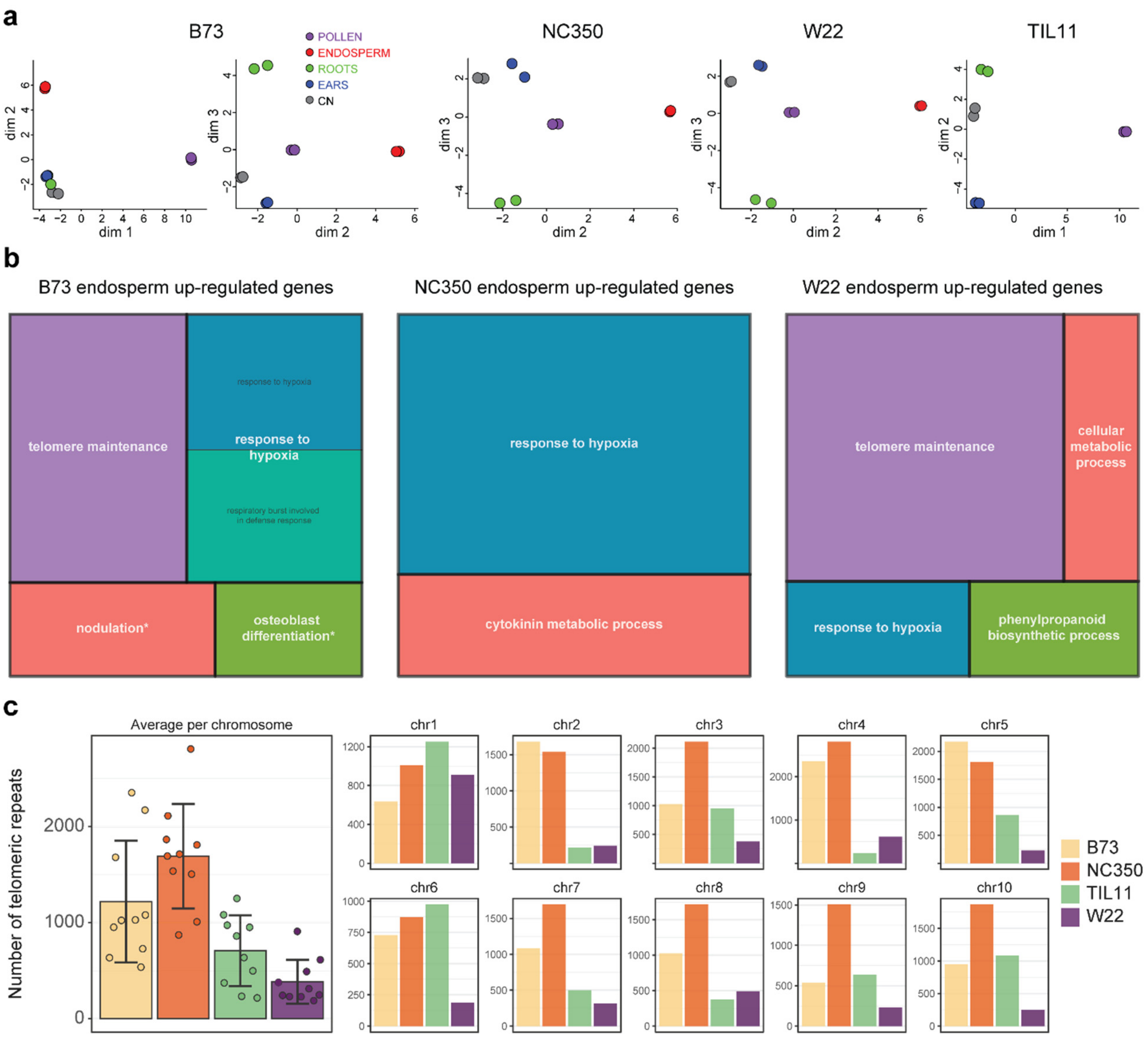

Supplementary Fig. 3. Similar tissue-specific transcriptional profiles in maize and teosinte inbreds.

a. Multidimensional scaling (MDS) plots of RNA-seq replicates in each of the 5 tissues of B73, NC350, W22 and the 4 tissues of TIL11. In all inbreds, biological replicates within samples cluster tightly together, while pollen and endosperm samples are most distinct. **b.** Gene ontology (GO) terms enriched in genes up-regulated in endosperm versus all other tissues of B73, NC350 and W22. Like pollen (see **Fig. 3b**), NC350 endosperm does not have an enrichment of genes involved in telomere maintenance. *'nodulation' and 'osteoblast differentiation' are restricted to legumes and vertebrates, and are errors of electronic annotation inference (IEA) in this context; the 5 genes containing these terms code for membrane proteins implicated in cellularization. **c**. Number of telomeric repeats ("CCCTAAA") present at the ends of each chromosome (within 1Mb) in each of the 4 assembled genomes. NC350 contains more copies than the other inbreds on average, potentially linked to the lack of differential expression of genes involved in telomere maintenance seen in **b** and in **Fig. 3b.**

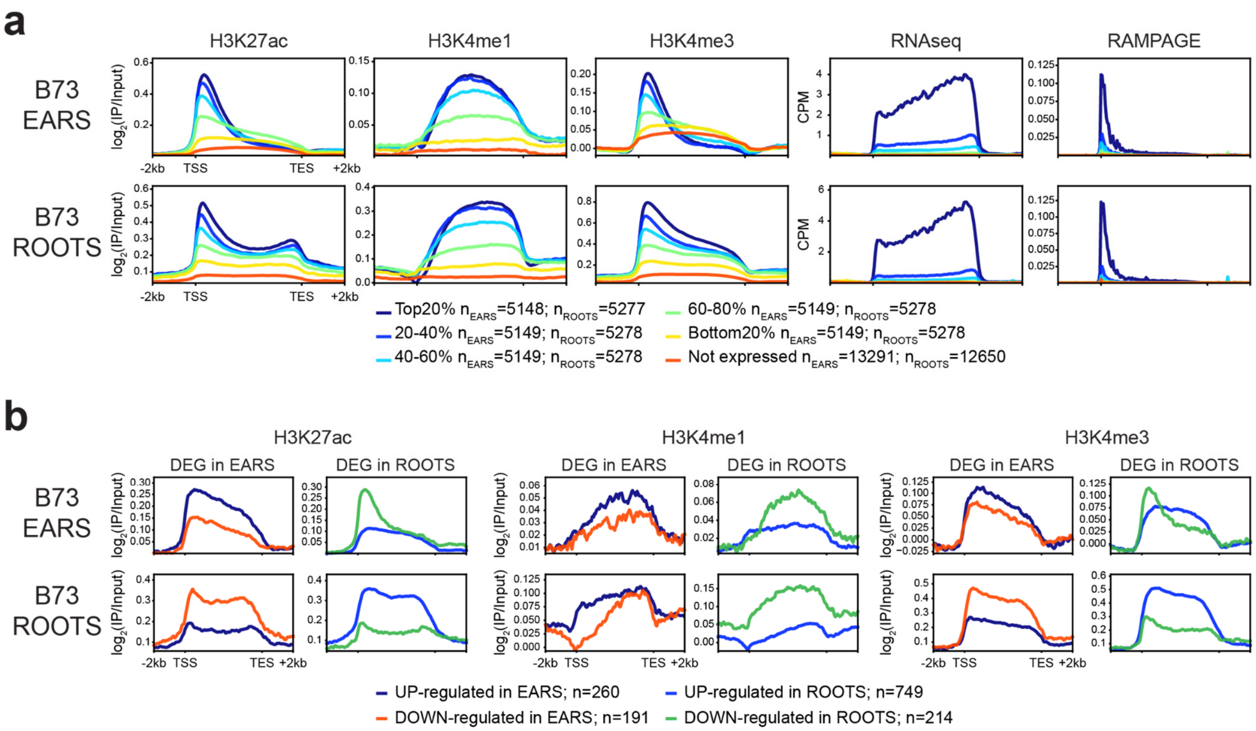

Supplementary Fig. 4. Histone modification levels correlate with gene expression within tissues, but H3K4me1 does not correlate with differential expression between tissues.
a. Metaplots of ChIP-seq, RNA-seq and RAMPAGE on genes grouped by expression levels within each tissue (RPKM). **b.** Metaplots of ChIP-seq in genes up or down-regulated in one tissue (immature ears or root tips) versus all other tissues.

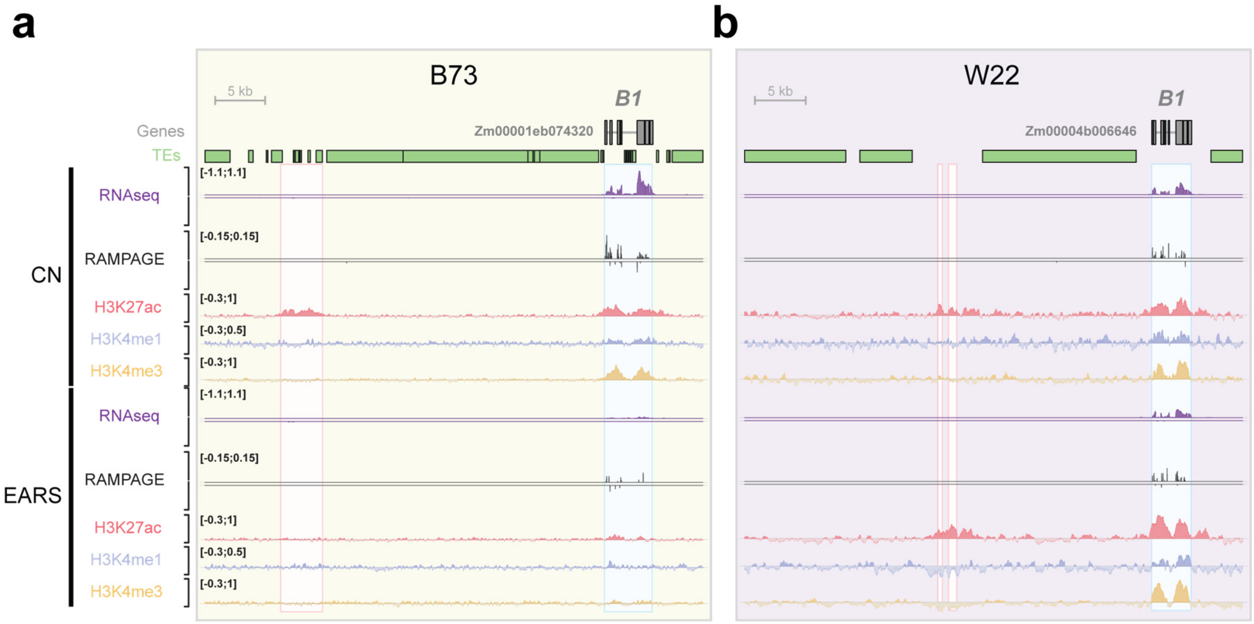

Supplementary Fig. 5. Example of tissue-specific gene regulation in two inbreds: the *Booster1* (*B1*) locus.

a, b. Browser shots of ChIP-seq (log_2[IP/Input]) and transcriptomic data (CPM) in the *Booster 1* (*B1*) locus in coleoptilar nodes (CN) and immature ears of B73 (**a**) and W22 (**b**). In B73, the enhancer (red box) is active in the CN but inactive in immature ears, as shown by the peak of H3K27ac correlated with expression of the gene (blue box). In W22, the gene is expressed in both tissues, and the enhancer has H3K27ac signal in both tissues, which recapitulates the difference in pigmentation between the two lines.

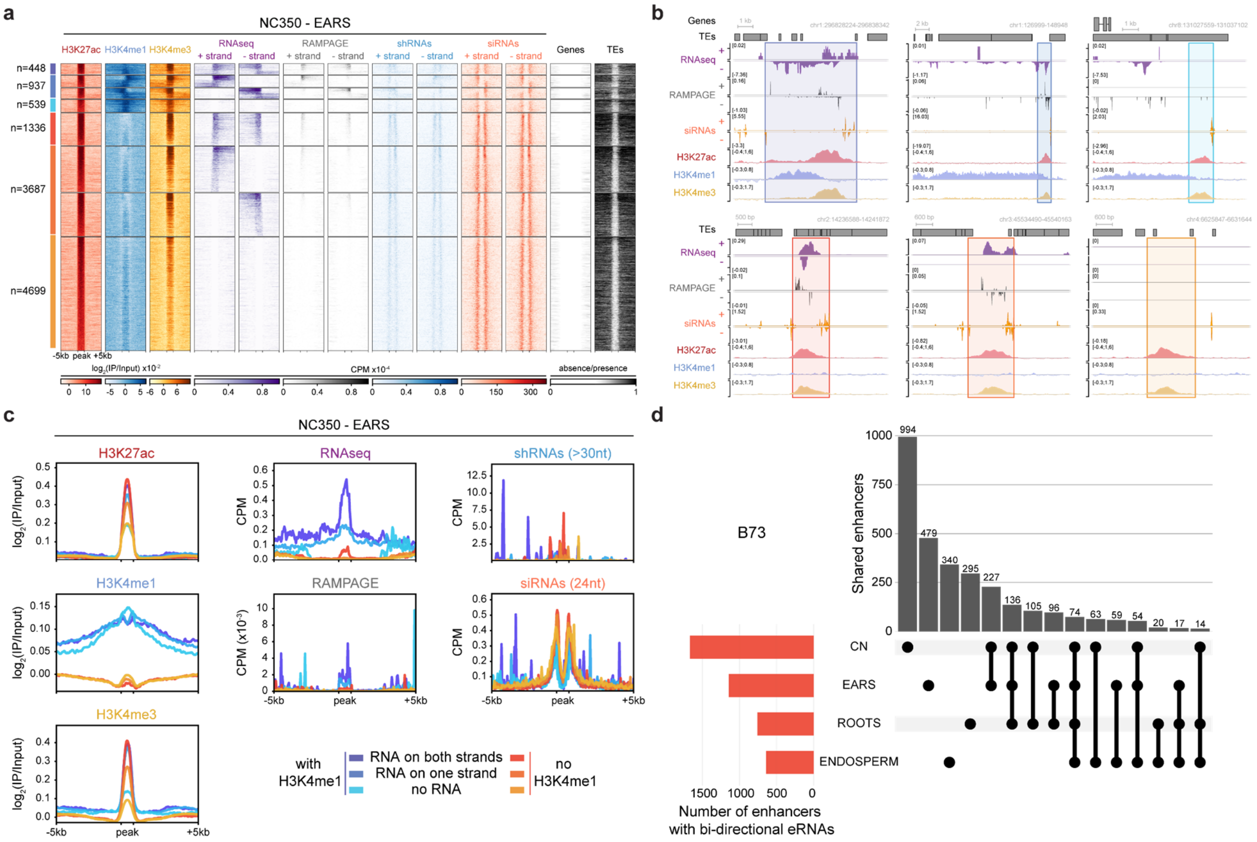

Supplementary Fig. 6. Enhancers with bi-directional enhancer RNAs are tissue-specific. **a.** Heatmap of ChIP-seq and transcriptomic datasets in NC350 immature ears at distal H3K27ac peaks. Six classes of regulatory regions were identified based on the presence (blue) or the absence (red) of H3K4me1 peaks within 1kb, and on the presence of RNA-seq reads mapping to both strands, one strand, or none (from darker to lighter shades). The short RNA-seq datasets were split into longer fragments (>30nt) labeled shRNA and canonical siRNAs (24nt). Presence (black) and absence (white) of annotated genes and TEs surrounding the peaks are shown, demonstrating the absence of annotated features within regulatory regions. **b.** Browser screenshots of representative examples of each class of H3K27ac peaks (boxed), with (upper) and without (lower) H3K4me1 peaks within 1kb. H3K4me1 peaks indicate the presence of unannotated genes. **c.** Metaplots of the data summarizing the heatmap from (**a**), with regions expressed on one strand being averaged together. **d.** Upset plot of the enhancers with bi-directional enhancer RNAs identified in the four tissues of B73. The total number of enhancers identified in each tissue is shown on the histogram on the left-hand side. The number of shared loci between the different tissues are shown above the intersection matrix.

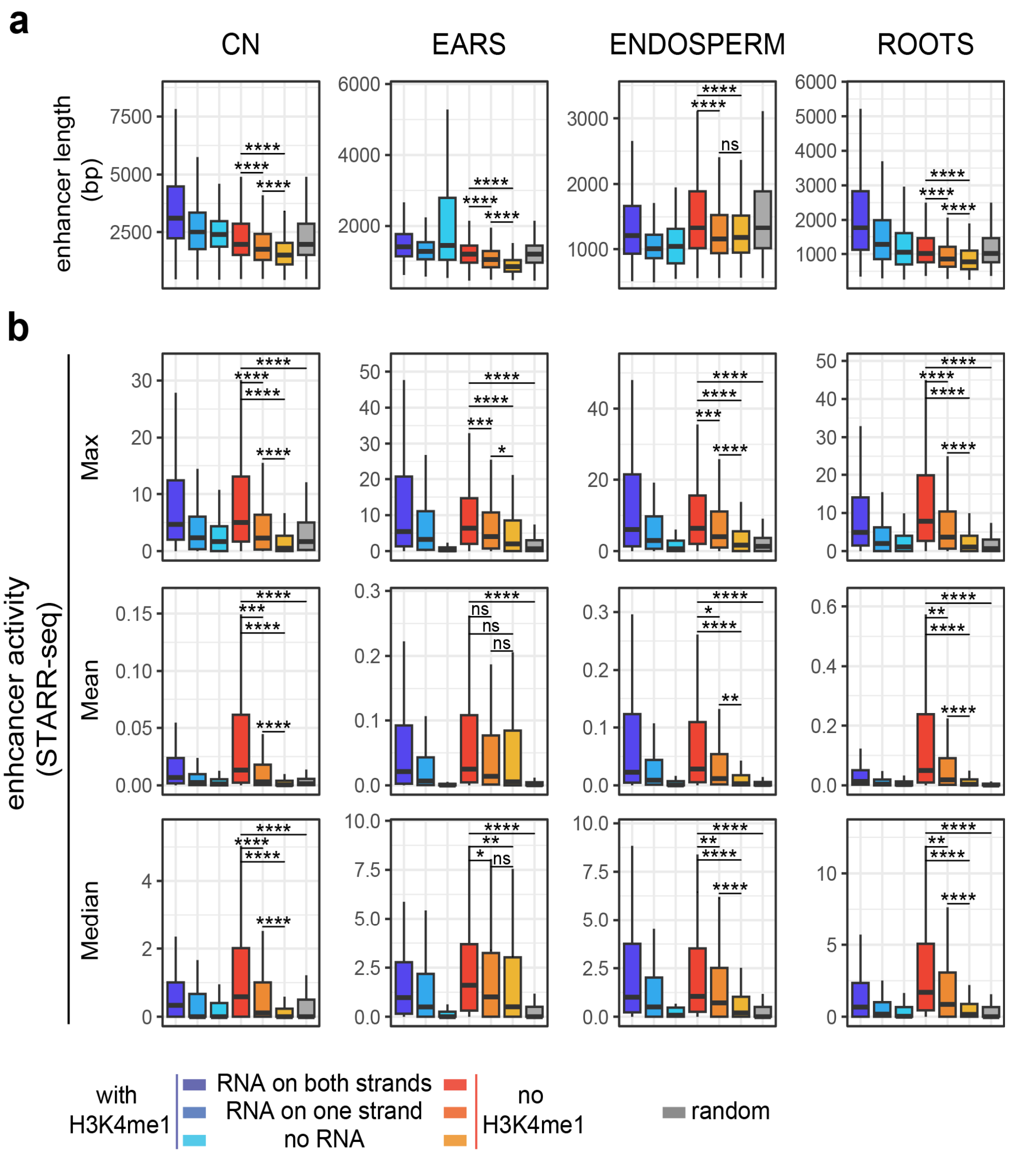

Supplementary Fig. 7. Expressed enhancers are longer and inherently drive more expression, in all tissues.

a. Size distribution of the distal H3K27ac peaks in each tissue of B73, split by the presence of H3K4me1 peak within 1kb, and the presence of RNA within the peaks. A set of control regions consisting of the bi-directional enhancers shuffled in the genome was included (two-sided t-test, * $p<10^{-2}$; ** $p<10^{-3}$; *** $p<10^{-4}$; **** $p<10^{-5}$; ns not significant). **b.** Distribution of enhancer activity of the same clusters than in (**a**), as measured by STARR-seq[4]. The maximum value found in each enhancer, the mean value of each enhancer, and the median value of each enhancer were plotted and always show higher activity of enhancers with bi-directional eRNAs (two-sided t-test, * $p<10^{-2}$; ** $p<10^{-3}$; *** $p<10^{-4}$; **** $p<10^{-5}$; ns not significant). Data shows distribution of max, mean or median STARR-seq value, respectively, at all distal H3K27ac peaks, with boxplots showing the mean and ranging from first to third quartiles, whiskers mark 1.5×IQR, and outliers are not shown. Source data are provided as a Source Data file.

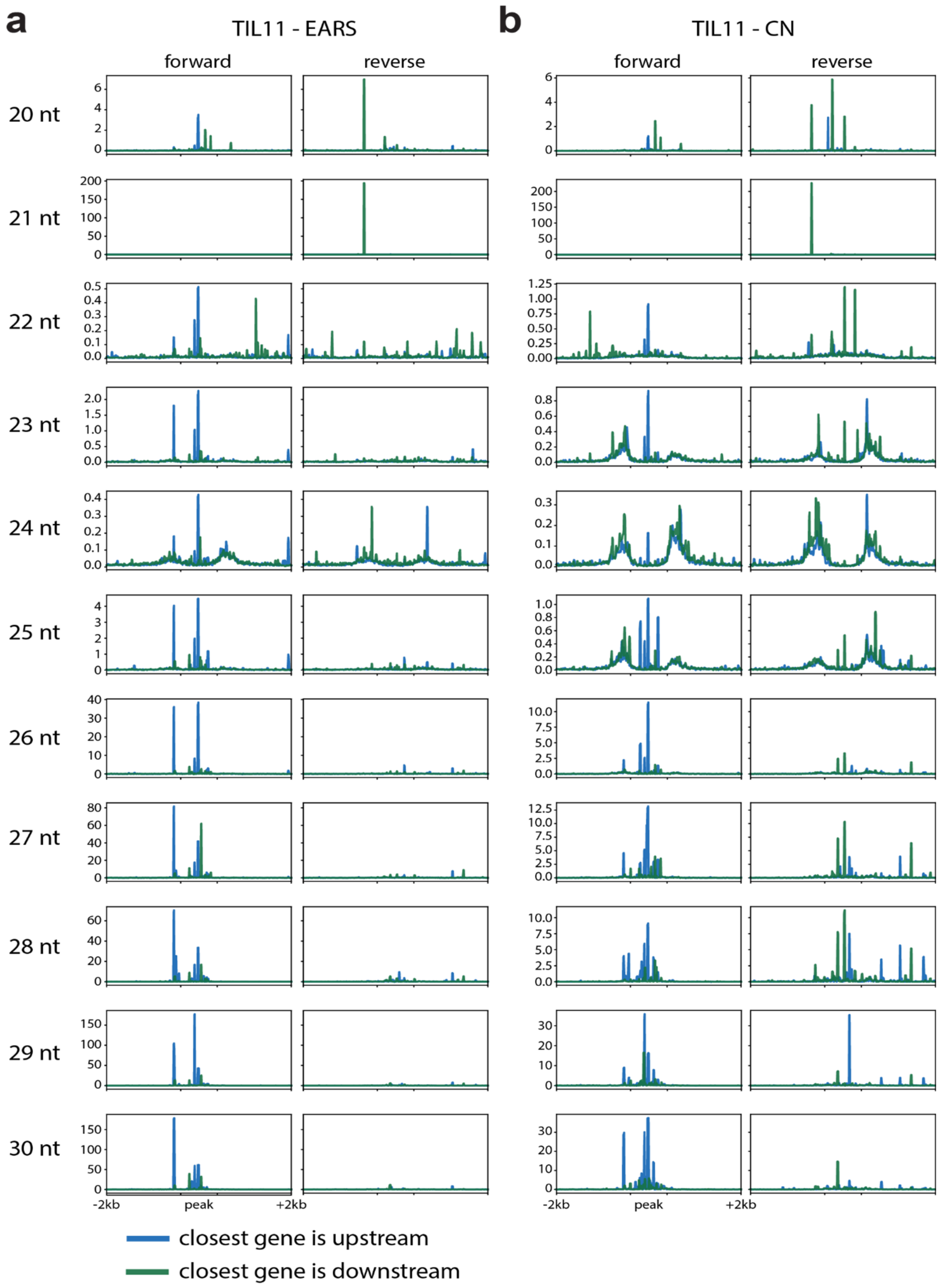

Supplementary Fig. 8. Small interfering RNAs also target boundaries of enhancers in TIL11. a,b. Metaplots of short RNA-seq from TIL11 immature ears (**a**) and coleoptilar nodes (CN) (**b**) at distal H3K27ac peaks identified in TIL11 immature ears. Small RNAs were split by read length from 20 to 30 nucleotides and mapping strands (forward or reverse) before normalization in count per million (CPM), and plotted on H3K27ac peaks which were further than 2kb from the closest gene, either upstream (green) or downstream (blue) from it.

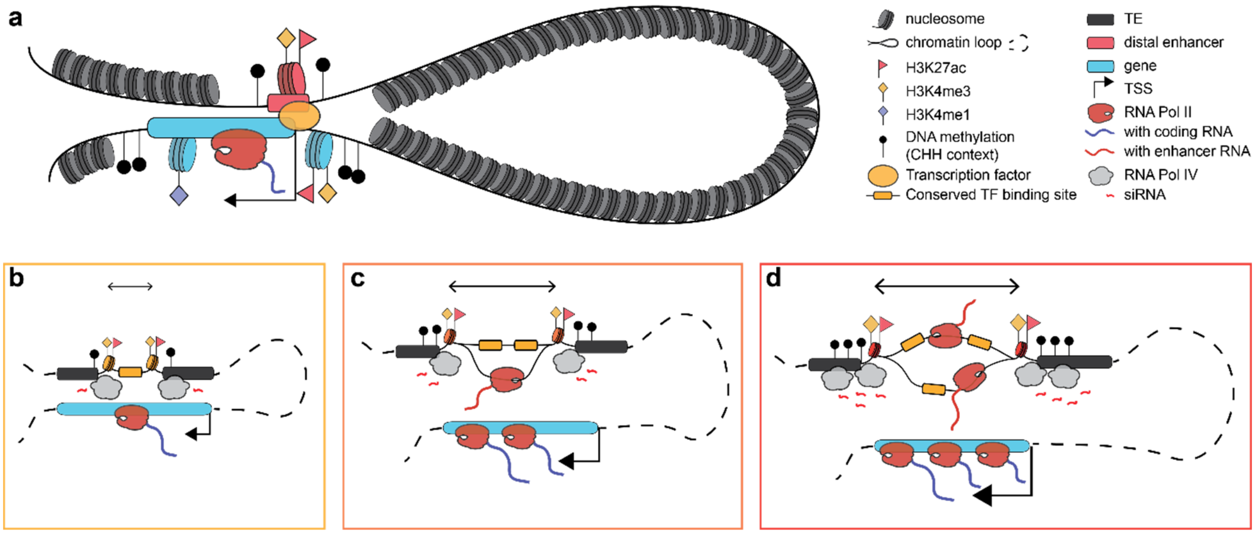

Supplementary Fig. 9. Model of enhancers with bi-directional enhancer RNAs.
a. Distal enhancers, marked with H3K27ac and H3K4me3, form chromatin loops with the genes they regulate through transcription factors recruiting RNA polymerase. Genes are also marked by H3K27ac and H3K4me3 at their promoter, and contain H3K4me1 along the gene body. Both genetic features are surrounded by transposable elements and have CHH DNA methylation (mCHH) deposited by the RdDM machinery at their boundaries. Enhancers with higher number of transcription factor binding sites promote higher gene expression (shown by more RNA PolII proteins and bigger TSS arrows) which is correlated with larger, more accessible regions (bigger horizontal arrow) and an increase in transcription of enhancer RNAs from: **b**, no enhancer RNA (eRNA); **c**, eRNA on one strand; and **d**, bi-directional eRNAs. This increase in accessibility is visible at the enhancer with an elevated level of H3K27ac and H3K4me3 (larger symbols), and higher RdDM activity and mCHH at the boundaries (more Pol IV producing siRNAs and black lollipops), protecting transcription to extend into the neighboring transposable elements.

Supplementary References

1. Turpin, Z. M. *et al.* Chromatin structure profile data from DNS-seq: Differential nuclease sensitivity mapping of four reference tissues of B73 maize (Zea mays L). *Data Brief* **20**, 358–363 (2018).
2. Xu, X. *et al.* Single-cell RNA sequencing of developing maize ears facilitates functional analysis and trait candidate gene discovery. *Dev. Cell* **56**, 557-568.e6 (2021).
3. Sun, Y. *et al.* 3D genome architecture coordinates trans and cis regulation of differentially expressed ear and tassel genes in maize. *Genome Biol.* **21**, 1–25 (2020).
4. Ricci, W. A. *et al.* Widespread long-range cis-regulatory elements in the maize genome. *Nature Plants* **5**, 1237–1249 (2019).
