# Supplementary material for: MaizeCODE reveals bi-directionally expressed enhancers that harbor molecular signatures of maize domestication
Source: Nat Commun. 2024 Dec 30;15:10854. doi: 10.1038/s41467-024-55195-w (PMC11685423; doi:10.1038/s41467-024-55195-w)
Supplement: Supplementary file 3 — Description of additional supplementary files [file 41467_2024_55195_MOESM3_ESM.pdf]

Description of Additional Supplementary files

Supplementary Data 1. List of differentially expressed genes in all pairwise comparisons. Each sheet corresponds to one inbred.

Supplementary Data 2. Metrics of all ChIPseq, RNA, RAMPAGE and short RNA datasets. The first few lines of each sheet contain descriptions of each column.
