# Supplementary material for: MaizeCODE reveals bi-directionally expressed enhancers that harbor molecular signatures of maize domestication
Source: Nat Commun. 2024 Dec 30;15:10854. doi: 10.1038/s41467-024-55195-w (PMC11685423; doi:10.1038/s41467-024-55195-w)
Supplement: Supplementary file 6 — Reporting Summary [file 41467_2024_55195_MOESM6_ESM.pdf]

nature portfolio

Corresponding author(s): Thomas Gingeras and Robert Martienssen

Last updated by author(s): Nov 1, 2024

Reporting Summary

Nature Portfolio wishes to improve the reproducibility of the work that we publish. This form provides structure for consistency and transparency in reporting. For further information on Nature Portfolio policies, see our Editorial Policies and the Editorial Policy Checklist.

Statistics

For all statistical analyses, confirm that the following items are present in the figure legend, table legend, main text, or Methods section.

n/a	Confirmed	
☐	☒	The exact sample size (*n*) for each experimental group/condition, given as a discrete number and unit of measurement
☐	☒	A statement on whether measurements were taken from distinct samples or whether the same sample was measured repeatedly
☐	☒	The statistical test(s) used AND whether they are one- or two-sided<br>*Only common tests should be described solely by name; describe more complex techniques in the Methods section.*
☐	☒	A description of all covariates tested
☐	☒	A description of any assumptions or corrections, such as tests of normality and adjustment for multiple comparisons
☐	☒	A full description of the statistical parameters including central tendency (e.g. means) or other basic estimates (e.g. regression coefficient) AND variation (e.g. standard deviation) or associated estimates of uncertainty (e.g. confidence intervals)
☐	☒	For null hypothesis testing, the test statistic (e.g. *F*, *t*, *r*) with confidence intervals, effect sizes, degrees of freedom and *P* value noted<br>*Give P values as exact values whenever suitable.*
☒	☐	For Bayesian analysis, information on the choice of priors and Markov chain Monte Carlo settings
☒	☐	For hierarchical and complex designs, identification of the appropriate level for tests and full reporting of outcomes
☒	☐	Estimates of effect sizes (e.g. Cohen's *d*, Pearson's *r*), indicating how they were calculated

Our web collection on statistics for biologists contains articles on many of the points above.

Software and code

Policy information about availability of computer code

Data collection	NA
Data analysis	All analyses are described in the Methods section of the manuscript, and the code is available on https://github.com/martienssenlab/maize-code. The following softwares were used: bedtools 2.29.2; bowtie2 64-bit 2.4.1; Compiler: gcc version 7.5.0 (crosstool-NG 1.24.0.131_87df0e6_dirty); cutadapt 2.10; deeptools 3.5.0; dplyr 1.0.6; fastqc 0.11.9; homer 4.11; IDR 2.0.4.2; bedSort; bedGraphToBigWig; macs2 2.2.7.1; meme 5.3.0; multiqc 1.11; sra-tools 2.11.0; pigz 2.3.4; samtools 1.10 (Using htslib 1.10.2); seqkit 0.13.2; shortstack 3.8.5; STAR 2.7.5c; wget 1.20.1; R 4.0.3 + R packages: dplyr 1.0.6; tidyr 1.1.3; ggplot2 3.3.5; cowplot 1.1.1; RColorBrewer 1.1-2; AnnotationForge 1.32.0; rrvgo 1.5.3; topGO 2.42.0; purrr 0.3.4; limma 3.46.0; edgeR 3.32.1; stringr 1.4.0; ComplexUpset 1.2.1

For manuscripts utilizing custom algorithms or software that are central to the research but not yet described in published literature, software must be made available to editors and reviewers. We strongly encourage code deposition in a community repository (e.g. GitHub). See the Nature Portfolio guidelines for submitting code & software for further information.

Data

Policy information about availability of data

All manuscripts must include a data availability statement. This statement should provide the following information, where applicable:

- Accession codes, unique identifiers, or web links for publicly available datasets
- A description of any restrictions on data availability
- For clinical datasets or third party data, please ensure that the statement adheres to our policy

Sequence data generated for this study have been deposited in Gene Expression Omnibus SuperSeries GSE254496. The maize genomes for B73, NC350 and W22 were downloaded from https://www.maizegdb.org/ and the genome and annotations for TIL11 were deposited to https://maize-pangenome.gramene.org/.

Research involving human participants, their data, or biological material

Policy information about studies with human participants or human data. See also policy information about sex, gender (identity/presentation), and sexual orientation and race, ethnicity and racism.

Reporting on sex and gender	NA
Reporting on race, ethnicity, or other socially relevant groupings	NA
Population characteristics	NA
Recruitment	NA
Ethics oversight	NA

Note that full information on the approval of the study protocol must also be provided in the manuscript.

Field-specific reporting

Please select the one below that is the best fit for your research. If you are not sure, read the appropriate sections before making your selection.

☒ Life sciences ☐ Behavioural & social sciences ☐ Ecological, evolutionary & environmental sciences

For a reference copy of the document with all sections, see nature.com/documents/nr-reporting-summary-flat.pdf

Life sciences study design

All studies must disclose on these points even when the disclosure is negative.

Sample size	No sample-size calculations were performed for this study. Sample size were chosen based on convention of the field per ENCODE (https://genome.cshlp.org/content/genome/22/9/1813.full.html)
Data exclusions	No data was excluded from the analysis.
Replication	Two biological replicates were performed for all experiments, and all attempts at replication were successful.
Randomization	Biological samples were taken randomly from growing plants. For bioinformatics analysis, a set of control regions were chosen by randomizing their distribution in the genome with bedtools shuffle.
Blinding	No blinding were required since only wild-type samples were used in this study. All analyses were performed using the same parameters for all tissues and inbred lines.

Reporting for specific materials, systems and methods

We require information from authors about some types of materials, experimental systems and methods used in many studies. Here, indicate whether each material, system or method listed is relevant to your study. If you are not sure if a list item applies to your research, read the appropriate section before selecting a response.

Materials & experimental systems

n/a	Involved in the study
☐	☒ Antibodies
☒	☐ Eukaryotic cell lines
☒	☐ Palaeontology and archaeology
☒	☐ Animals and other organisms
☒	☐ Clinical data
☒	☐ Dual use research of concern
☐	☒ Plants

Methods

n/a	Involved in the study
☐	☒ ChIP-seq
☒	☐ Flow cytometry
☒	☐ MRI-based neuroimaging

Antibodies

Antibodies used	anti-H3K4me1 (Abcam, ab8895); anti-H3K4me3 (Millipore, 07-473); anti-H3K27ac (Abcam, ab4729); high-affinity GFP-Trap magnetic agarose (ChromoTek, gtma-20)
Validation	Antibodies are ChIP-grade and tested by the manufacturer.

Dual use research of concern

Policy information about dual use research of concern

Hazards

Could the accidental, deliberate or reckless misuse of agents or technologies generated in the work, or the application of information presented in the manuscript, pose a threat to:

No	Yes
☒	☐ Public health
☒	☐ National security
☒	☐ Crops and/or livestock
☒	☐ Ecosystems
☒	☐ Any other significant area

Experiments of concern

Does the work involve any of these experiments of concern:

No	Yes
☒	☐ Demonstrate how to render a vaccine ineffective
☒	☐ Confer resistance to therapeutically useful antibiotics or antiviral agents
☒	☐ Enhance the virulence of a pathogen or render a nonpathogen virulent
☒	☐ Increase transmissibility of a pathogen
☒	☐ Alter the host range of a pathogen
☒	☐ Enable evasion of diagnostic/detection modalities
☒	☐ Enable the weaponization of a biological agent or toxin
☒	☐ Any other potentially harmful combination of experiments and agents

Plants

Seed stocks | Seeds stocks for B73, W22 and NC350 were obtained from the Maize Genetics Stock Center, and for TIL11 from Dr. John Doebley.

Novel plant genotypes | No novel genotypes were generated in this study.

Authentication | NA

ChIP-seq

Data deposition

☒ Confirm that both raw and final processed data have been deposited in a public database such as GEO.

☒ Confirm that you have deposited or provided access to graph files (e.g. BED files) for the called peaks.

Data access links<br>*May remain private before publication.*	https://www.ncbi.nlm.nih.gov/geo/query/acc.cgi?acc=GSE254496
Files in database submission	Raw sequencing data and called peaks for: 2 replicates of H3K4me1, H3K4me3 and H3K27ac IPs and Inputs for each tissue (ears, cn, roots, endosperm) for B73, W22 and NC350; 2 replicates of H3K27ac IP and Inputs for TIL11 ears; 2 biological replicates for GT1-YFP and TU1A-YFP IPs and corresponding inputs.
Genome browser session<br>(e.g. UCSC)	https://maize-pangenome-ensembl.gramene.org/

Methodology

Replicates	2 biological replicates per sample.
Sequencing depth	A minimum of 10M reads and an average of 50M per replicate.
Antibodies	anti-H3K4me1 (Abcam, ab8895); anti-H3K4me3 (Millipore, 07-473); anti-H3K27ac (Abcam, ab4729); high-affinity GFP-Trap magnetic agarose (ChromoTek, gtma-20)
Peak calling parameters	Peaks were called comparing the IP to the corresponding Input with macs2 default parameters, using the following command for narrow peaks for H3K27ac, H3K4me3 and TFs:<br>macs2 callpeak -t ${namefiletype} -c ${inputfiletype} -f BAMPE -g 2.2e9 ${param} -n ${name}_${filetype} --keep-dup "all" --call-summits --outdir peaks/ --tempdir $TMPDIR<br>and this command for broad peaks for H3K4me1:<br>macs2 callpeak -t ${namefiletype} -c ${inputfiletype} -f BAMPE -g 2.2e9 ${param} -n ${name}_${filetype} --keep-dup "all" --outdir peaks/ --tempdir $TMPDIR --broad
Data quality	Data quality was assessed by mapping statistics, correlation between replicates and comparison to previously reported datasets. Only peaks present in merged replicates and two pseudo-replicates were retained.
Software	ChIPseq was analyzed as described in the Methods with the code deposited at https://github.com/martienssenlab/maize-code. Bowtie2 was used to map reads, MACS2 was used to call peaks.
